# Radiosynthesis and preclinical evaluation of [^11^C]SNX-ab as an Hsp90α,β isoform-selective PET probe for in vivo brain and tumour imaging

**DOI:** 10.1186/s41181-023-00189-0

**Published:** 2023-01-30

**Authors:** Romy Cools, Koen Vermeulen, Valeria Narykina, Renan C. F. Leitao, Guy Bormans

**Affiliations:** 1 Laboratory for Radiopharmaceutical Research, Department of Pharmaceutical and Pharmacological Sciences, KU Leuven, 3000 Leuven, Belgium; 2 NURA, Belgian Nuclear Research Centre (SCK CEN), 2400 Mol, Belgium; 3 Switch Laboratory, VIB Center for Brain and Disease Research, Herestraat 49, 3000 Leuven, Belgium; 4 Switch Laboratory, Department of Cellular and Molecular Medicine, KU Leuven, Herestraat 49, 3000 Leuven, Belgium

**Keywords:** Hsp90α/β isoform, PET, Carbon-11, Brain, Neurodegenerative disorders, Tumour

## Abstract

**Background:**

The molecular chaperone, Hsp90, is a key player in the protein quality control system that maintains homeostasis under cellular stress conditions. It is a homodimer with ATP-dependent activity, and is a prominent member of the chaperone machinery that stabilizes, matures and (re)folds an extensive list of client proteins. Hsp90 occurs as four isoforms, cytosolic Hsp90α and Hsp90β, mitochondrial TRAP1 and Grp94 present in the endoplasmic reticulum. An aberrant role of Hsp90 has been attributed to several cancers and neurodegenerative disorders. Consequently, Hsp90 has emerged as an attractive therapeutic target. However, pan-Hsp90 inhibition often leads to detrimental dose-limiting toxicities. Novel strategies for Hsp90-targeted therapy intend to avoid this by using isoform-specific Hsp90 inhibition. In this respect, the radiosynthesis of carbon-11 labeled SNX-ab was developed and [^11^C]SNX-ab was evaluated as a Hsp90α,β isoform-selective PET probe, which could potentially allow to quantify in vivo Hsp90α,β expression.

**Results:**

[^11^C]SNX-ab was synthesized with excellent radiochemical yields of 45% and high radiochemical purity (> 98%). In vitro autoradiography studies on tissue slices of healthy mouse brain, mouse B16.F10 melanoma and U87 glioblastoma using homologous (SNX-ab, SNX-0723) and heterologous (Onalespib and PU-H71) Hsp90 inhibitors demonstrated only limited reduction of tracer binding, indicating that the binding of [^11^C]SNX-ab was not fully Hsp90-specific. Similarly, [^11^C]SNX-ab binding to U87 cells was not efficiently inhibited by Hsp90 inhibitors. *Ex vivo* biodistribution studies in healthy mice revealed limited brain exposure of [^11^C]SNX-ab and predominantly hepatobiliary clearance, which was confirmed by in vivo full-body dynamic µPET studies.

**Conclusion:**

Our results suggest that [^11^C]SNX-ab is not an ideal probe for in vivo visualization and quantification of Hsp90α/β expression levels in tumour and brain. Future research in the development of next-generation Hsp90 isoform-selective PET tracers is warranted to dissect the role played by each isoform towards disease pathology and support the development of subtype-specific Hsp90 therapeutics.

**Supplementary Information:**

The online version contains supplementary material available at 10.1186/s41181-023-00189-0.

## Background

Molecular chaperones are critical players in the protein quality control system to buffer cellular stress and guarantee cellular homeostasis. The 90 kDa chaperone, heat shock protein 90 (Hsp90), occurs as four paralogues found in the cytoplasm (Hsp90α,β), endoplasmic reticulum (ER) (94 kDa glucose-regulated protein (Grp94)) and mitochondria (tumour necrosis factor receptor-associated protein 1 (TRAP1)) and can adopt several conformations (Hoter et al. 2018). Hsp90 interacts with a multitude of co-chaperones, other Hsps and clients to establish the chaperome, a transient dynamic complex that stabilizes, matures and refolds misfolded proteins, and regulates protein trafficking and signal transduction (Streicher 2019). Hsp90 functions as a dimer and is ATP-dependent.

A variety of diseases that cause or result from proteotoxic stress are associated with Hsp90 function. Hsp90 expression is upregulated in several human tumours, including breast, ovary and prostate cancer, glioblastoma, melanoma and hepatocellular carcinomas (Lianos et al. 2015). In addition, cancer cells are particularly sensitive to Hsp90 inhibitors (Miyata et al. 2013), possibly attributed to post-translational modifications (Backe et al. 2020), conformational changes of Hsp90 (Beebe et al. 2013) and/or an increased degree of connectivity within the chaperome network, termed the epichaperome. This suggests that tumour Hsp90 is present in a different state and plays a deviant role by the folding/stabilization of multiple onco-proteins. The epichaperome is observed in chronic cellular stress situations, including cancer, but also neurodegenerative diseases (Joshi et al. 2018; Kishinevsky et al. 2018; Rodina et al. 2016).

Neurodegenerative diseases are per definition proteinopathies, driven by the aggregation of proteins, causing proteotoxic stress. Hsp90, several co-chaperones (CHIP, Hop, Aha1, Cdc37, FKBP51, p23, etc.) and client proteins of Hsp90 have been implied in neurodegenerative disease onset and progression ﻿(Koopman and Rüdiger 2020; Takeuchi et al. 2017), including Alzheimer’s disease (AD), Parkinson’s disease (PD) and Huntington’s disease (HD). Consequently, upregulation of Hsp90 in central nervous system (CNS) disorders has been reported to occur similarly to cancer pathology. In these disorders, the Hsp90 system is claimed to play an aberrant role, by preventing the proteasomal degradation of defective proteins, thereby sustaining the accumulation of aggregates and maintaining the malignant state (Ernst et al. 2014b; Inda et al. 2020; Kandratavicius et al. 2014; Luo et al. 2010; Sarah Kishinevsky and Wenjie Lou 2013; Takeuchi et al. 2017).

**Fig. 1:**
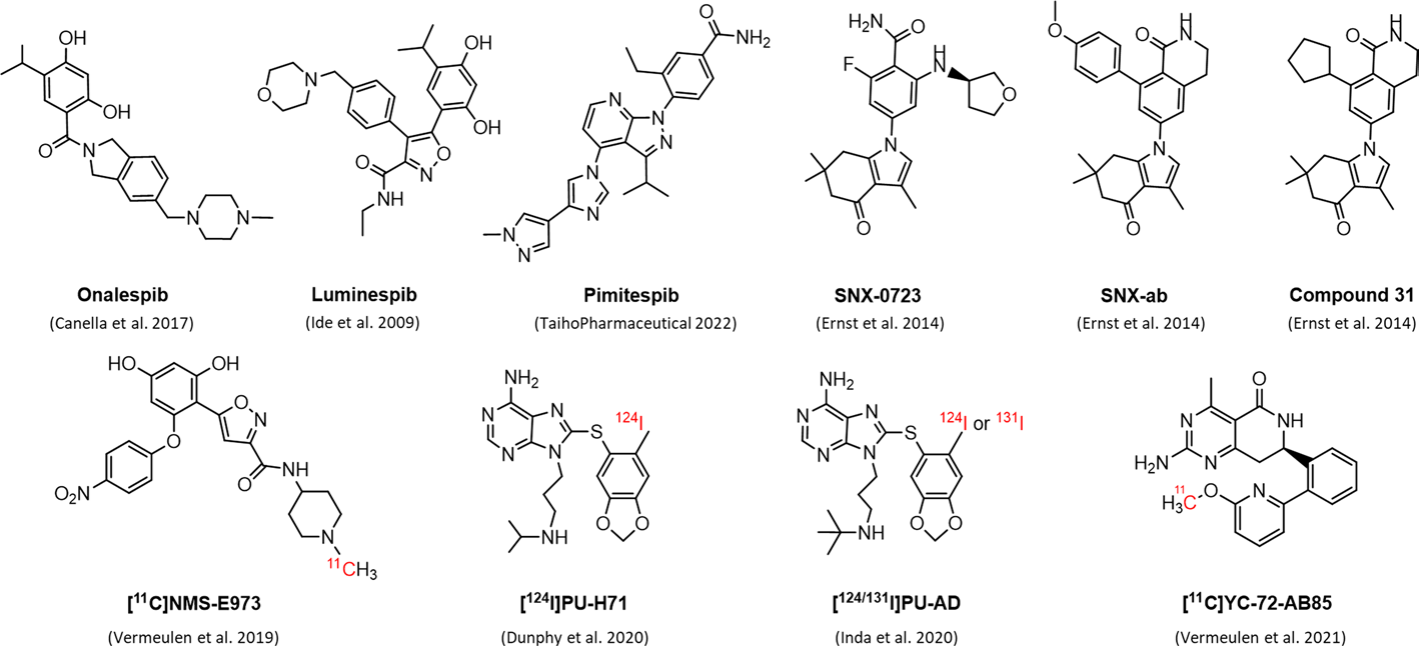
Chemical structures of Hsp90 inhibitors

Multiple promising Hsp90 inhibitors that have entered clinical trials, including Onalespib (Canella et al. 2017) and Luminespib (Ide et al. 2009; Sessa et al. 2009) (Fig. 1), which to date have been primarily focused on treating oncology indications, suffer from poor pharmacokinetic profiles, induction of the heat shock response (HSR) leading to resistance, and adverse effects including cardio, hepatic, and ocular toxicities, as well as dose-limiting toxicities (Yuno et al. 2018).

Currently, efforts are being put forth to develop Hsp90 isoform-selective compounds as a strategy to reduce these mechanism-based toxicities associated with pan-Hsp90 inhibition by selectively targeting the cytosolic isoforms of Hsp90 (Hsp90α and/or β), while not affecting the mitochondria (TRAP1) and ER (Grp94) isoforms in order to retain the essential Hsp90 functions for ER and mitochondrial viability and improve clinical outcomes (Li et al. 2020; Sanchez et al. 2019). Recently, the oral Hsp90α/β isoform-specific inhibitor Pimitespib (Tas-116) (Fig. 1) was registered in Japan as the first Hsp90 drug for treating gastrointestinal stromal tumours (TaihoPharmaceutical 2022).

In this respect, Ernst et al. (2014b) applied a structure-based drug design strategy to develop a series of novel Hsp90α/β isoform-specific inhibitor compounds as second generation derivatives of the brain-permeable orally active compound SNX-0723 (Fig. 1, Table 1), which possesses a modest degree of Hsp90α/β isoform selectivity. A new series of potent, benzolactam structure-based, Hsp90α/β selective inhibitors were identified and evaluated in human HD patient-derived fibroblasts. A selected number of promising compounds were evaluated in pharmacokinetic studies in rats to evaluate plasma clearance and brain exposure, MDR1-MDCK permeability assays to assess P-gp affinity and in vitro metabolite identification studies using human liver microsomes. It was concluded that the proposed selective Hsp90α/β inhibitors were equipotent to pan-Hsp90 inhibitors SNX-0723 and Luminespib in promoting the clearance of mutant huntingtin protein (mHtt) in vitro, yet with less cellular toxicity. The most isoform-selective compound of the series (compound 31, Fig. 1) additionally successfully crossed the blood–brain barrier (BBB) and reduced brain Htt levels following oral dosing in rats. This demonstrated in vivo proof-of-concept that selective Hsp90α/β inhibition with a small molecule could affect brain Htt levels and hence supports the continued study of the development of Hsp90α/β isoform-selective therapeutics as a strategy to treat HD and other CNS disorders.

**Table 1:** Physicochemical parameters, in vitro inhibitory concentrations and isoform selectivity profile of Hsp90 inhibitors used in this study

Compound	MW^a^	LogP	*t*PSA (Å^2^)^b^	IC_50_ (nM)	Hsp90α K_i_ (nM)	Hsp90β K_i_ (nM)	Grp94 K_i_ (nM)	TRAP1 K_i_ (nM)
SNX-ab	428.53	4.25	58.64	–	40 Ernst et al. (2014b)	40 Ernst et al. (2014b)	> 10 000 Ernst et al. (2014b)	> 10 000 Ernst et al. (2014b)
SNX-0723	399.47	1.84	84.66	14 Putcha et al. (2010)	3 Ernst et al. (2014a)	4 Ernst et al. (2014a)	375 Ernst et al. (2014a)	1195 Ernst et al. (2014a)
Onalespib	409.52	3.00	67.25	18 Woodhead et al. (2010)	–	–	–	–
PU-H71	512.37	4.38	96.83	51 Caldas-Lopes et al. (2009)	–	–	–	–

^a^*MW* Molecular weight, ^b^*tPSA* Topological polar surface area

Positron emission tomography (PET) with a relevant Hsp90 probe can aid in clinical investigation and Hsp90 inhibitor development by allowing the study of Hsp90 (isoform) occupancy, disease follow-up and therapeutic outcome. Several pan-selective Hsp90-targeting PET probes have been reported. [^11^C]NMS-E973 (Fig. 1) revealed Hsp90-specific binding to B16.F10 melanoma cells and to different tumour tissue slices. An in vivo µPET study confirmed Hsp90-specific tumour binding to B16.F10 melanoma inoculated mice and additionally demonstrated Hsp90-specific binding in blood, lungs and spleen of the tumour-bearing animals (Vermeulen et al. 2019). [^124^I]PU-H71 (Fig. 1) showed retention in MDA-MB-468 tumour mouse xenografts (high epichaperome levels), and the probe was subjected to clinical trials to study in vivo biodistribution, pharmacokinetics, metabolism and safety profile in breast or lymphoma cancer patients. The study showed some potential for epichaperome-targeted tumour imaging, although considerable variability in tracer retention among patients was observed (Dunphy et al. 2020).

Hsp90 PET probes that are suitable for brain imaging are limited. The Hsp90 probe [^131^I]PU-AD (Fig. 1) was used to visualize the epichaperome in a PS19 AD mouse model using autoradiography and its increased binding was found to precede tau fibril formation in the hippocampus (Inda et al. 2020). In AD patients, static PET images obtained 3 h after injection of [^124^I]PU-AD showed higher binding in the brain compared to the age-matched controls, yet no quantification of the PET results was reported (Inda et al. 2020). Recently, our group developed and evaluated [^11^C]YC-72-AB85 (Fig. 1) as an Hsp90 PET probe in B16.F10 melanoma bearing mice, healthy rodents and a non-human primate (Vermeulen et al. 2021). The study demonstrated the presence of major in vivo saturable pools of Hsp90 inhibitor binding in tumour tissue, blood and brain in healthy animals. The observed saturable blood pool binding in rats and a monkey was attributed to the blood cell fraction and, additionally, blood binding of the tracer was increased in diseased conditions compared to the healthy controls. This saturable Hsp90 binding pool in blood, of which the binding capacity may be increased in disease, functions as a sink for Hsp90 inhibitors, and hence considerably affects their pharmacokinetics and pharmacodynamics.

These promising preclinical results spark the development of second-generation Hsp90 subtype-specific PET probes to acquire insight into the contributions of the different Hsp90 isoforms to these saturable binding pools in health and disease, thereby accelerating and supporting the development of subtype-specific Hsp90 therapeutics. In this respect, we envisioned the development and evaluation of a Hsp90α/β subtype-specific carbon-11 labelled PET probe for visualization of the Hsp90α/β binding pool in the brain and tumour tissue. From the list of benzolactam-based selective Hsp90α/β inhibitors described by Ernst et al. (2014b), SNX-ab (Fig. 1, Table 1) with very similar isoform selectivity and cellular potency as compound 31 was selected because its structure was most suitable for straightforward carbon-11 labeling. The radiosynthesis of [^11^C]SNX-ab was developed and the tracer was evaluated as a tumour and brain PET imaging probe.

## Methods

### Reagents and chemicals

Synthesis of the reference compound, 8-(4-methoxyphenyl)-6-(3,6,6-trimethyl-4-oxo-4,5,6,7-tetrahydro-1H-indol-1-yl)-3,4-dihydroisoquinolin-1(2*H*)-one (4) and precursor compound, 8-(4-hydroxyphenyl)-6-(3,6,6-trimethyl-4-oxo-4,5,6,7-tetrahydro-1*H*-indol-1-yl)-3,4-dihydroisoquinolin-1(2*H*)-one (9), was performed as described by Ernst et al. (2014b) and a detailed synthetic protocol and characterization is provided in Supplementary Material (Additional file 1: Figs. S1–S11). Hsp90 inhibitors, Onalespib and PU-H71 were purchased from commercial suppliers Bio-Connect Life Sciences, Selleckchem or MedChem Express and used without further purification. SNX-0723 ((S)-2-fluoro-6-((tetrahydrofuran-3-yl)amino)-4-(3,6,6-trimethyl-4-oxo-4,5,6,7-tetrahydro-1H-indol-1-yl)benzamide) was synthesized as previously reported (Huang et al. 2009). Chemical structures and corresponding total polar surface area (tPSA) and LogP values were drawn/calculated using ChemDraw Ultra 15.0 (Perkin Elmer).

### Liquid chromatography-mass spectrometry (LC–MS) analysis

Intermediate reaction products were characterized using a Dionex Ultimate 3000 LC system (Thermo Fisher Scientific, Sunnyvale, USA) coupled to a high-resolution time-of-flight mass spectrometer (MaXis Impact, Bruker, Bremen, Germany) equipped with an orthogonal electrospray interface. Acquisition and processing of data were performed using Compass IsotopePattern (version 3.2, Bruker).

### Nuclear magnetic resonance (NMR) analysis

NMR was performed using proton nuclear magnetic resonance (^1^H NMR) at 400–600 MHz. Chemical shifts are reported in parts per million relative to tetramethylsilane (*δ* = 0). Carbon nuclear magnetic resonance (^13^C NMR) spectra were acquired at 101 MHz on a Bruker AVANCE 400–600 MHz spectrometer (5 mm probe, Bruker AG, Fällanden, Switzerland).

### High-performance liquid chromatography (HPLC) analysis

HPLC was performed on a LaChrom Elite HPLC system (Hitachi, Darmstadt, Germany) connected to a Waters 2487 UV–VIS detector and a 3-inch NaI(Tl) scintillation detector connected to a single channel analyser (Gabi, Raytest, Straubenhardt, Germany). Registration and integration of the HPLC chromatograms was performed with GINA Star (Raytest) software.

### Animals

Animals were kept in a thermoregulated (22 °C) and humidity-controlled environment with a 12 h/12 h light/dark cycle in individual ventilated cages and had free access to food and water. All animal experiments were conducted after approval of the local University Ethics Committee for Animals and according to the Belgian code of practice for the care and use of animals. Female C57BL/6 mice, 5–8 weeks of age (body mass 20–25 g) were purchased from Janvier (La Genest-Saint Isle, France). Female animals were chosen as they tend to grow less quickly and are generally more docile in nature. Animals were allowed to acclimatize for at least one week before start of the experiments.

### Preparation of tumour xenografts for tumour tissue

1*10^6^ U87 cells mixed with Cultrex (1:1; Cultrex Basement Membrane Extract, R&D systems, Minneapolis, MN, USA) were implanted subcutaneously into the right shoulder of female 10 week-old SCID mice (CB17.Cg-Prkdc < scid > Lyst < bg-J > /Crl; Charles River Laboratories, Sulzfeld, Germany). The tumours were allowed to grow for 4–5 weeks until they reached 150–200 mm^3^ in size (measured by caliper, h × l × w). 5*10^5^ B16.F10 cells mixed with Cultrex (1:1; Cultrex Basement Membrane Extract, R&D systems, Minneapolis, MN, USA) were implanted subcutaneously into the right shoulder of 8 week-old C57BL/6 mice (Janvier, La Genest-Saint Isle, France). The tumours were allowed to grow for 2–3 weeks until they reached 150–200 mm^3^ in size (measured by caliper, h × l × w).

### Preparation tumour and brain tissue for autoradiography

Tumour xenografts/healthy mice were anesthetized with 2.5% isoflurane in O_2_ at a flow rate of 1 L/min after which they were sacrificed by decapitation. Tumour/brain tissue was excised, rinsed with saline to remove blood and rapidly frozen in cooled 2-methylbutane (− 40 °C). Cryotome sectioning (Shandon cryotome FSE; Thermo Fisher, Waltham, MA) was performed to obtain 20 µm-sections. Tumour tissue slices were sectioned centrally and mouse brain sections were sectioned horizontally starting from 4.96 mm interaural and − 5.04 mm bregma to 3.16 and − 6.84, respectively. Sections were fixed on adhesive microscope slides (Superfrost Plus; Thermo Fisher Scientific) and stored at − 20 °C.

### Cell culture

The U87 cell line was obtained from American Type Culture Collection (ATCC). The cell line was grown according to the supplier’s instruction in DMEM medium (Dulbecco's Modified Eagle's Medium, high glucose, Gibco 500 mL (Fischer Scientific: 41965-039)) supplemented with 10% fetal bovine serum (FBS), 1X MEM non-essential amino acids (Gibco, Fischer Scientific: 11140-035) and 1 mM Sodium Pyruvate (Gibco, Fischer Scientific: 11360-039). Cell passage was kept under 20 for all experiments performed in this study.

### Quantification of radioactivity in biological samples

Quantification of radioactivity was performed with an automated gamma counter equipped with a 3-inch NaI(Tl) well crystal coupled to a multichannel analyzer (Wallac 1480 Wizard, Wallac, Turku, Finland). The results were corrected for background radiation, physical decay during counting and detector dead time.

### Radiosynthesis

Carbon-11 was produced by proton irradiation of a N_2_ + H_2_ (5%) gas mixture in a Cyclone 18/9 cyclotron (IBA Louvain-la-Neuve, Belgium) and obtained as [^11^C]CH_4_ by a ^14^ N(p,α)^11^C nuclear reaction. A home-built gas phase recirculation module was used for the conversion of [^11^C]CH_4_ to [^11^C]CH_3_I. The synthesis of [^11^C]SNX-ab was performed by bubbling of [^11^C]CH_3_I with a helium flow through a solution of the corresponding phenol SNX-ab precursor (250–300 µg) and Cs_2_CO_3_ (2.5–3 mg) dissolved in anhydrous dimethylformamide (DMF) (200–250 µL) at 100 °C for 3 min. After cooling down, the crude mixture was diluted with 1.3 mL of water and the reaction mixture was purified by HPLC on a RP-C_18_ column (XBridge C_18_ column, 5 µm, 4.6 mm × 150 mm; Waters, Milford, USA) eluted with 60/30 Na_2_HPO_4_ 0.01 M pH 9.3/ACN at a flow rate of 1.5 mL/min. The preparative HPLC collected fraction of [^11^C]SNX-ab is diluted with water and passed over a single use C_18_ Sep-Pak cartridge (activated with 5 ml ethanol 100% and 10 mL water). The Sep-Pak is then rinsed with 10 mL of water. [^11^C]SNX-ab is eluted from the Sep-Pak with approximately 1.1 mL ethanol into the final product sterile vial through a single-use sterile syringe filter (Millex-GV filter 0.22 µm, ø 13 mm Millipore, Billerica, MA). Next, a volume of approximately 11.9 mL NaCl 0.9% water for injection is rinsed over the Sep-Pak and filtered via the same filter into the final sterile vial to obtain a final EtOH concentration < 10%. A sample (100 µL) is taken for quality control (QC) analysis by HPLC on a RP-C_18_ column (XBridge C_18_ column, 3.5 µm, 3.0 mm × 100 mm; Waters, Milford, USA) eluted with 64/36 NaOAc 0.05 M pH 5.8/ACN at a flow rate of 0.6 mL/min to assess chemical and radiochemical purity. The column effluent was passed through a UV detector (254 nm) and a NaI(Tl) scintillation radioactivity detector as described in the HPLC analysis section. The identity of the tracer was confirmed by co-injection with authentic reference compound, SNX-ab, on the same HPLC system.

### In vitro autoradiography

The frozen tissue slices were air-dried and submerged in tris(hydroxymethyl)aminomethane hydrochloride (tris.HCl) 50 mM pH 7.4 for 10 min at room temperature. Next, the slices were air dried and pre-incubated with 200–300 µL tris.HCl 50 mM pH 7.4 + 0.3% BSA supplemented with dimethylsulfoxide (DMSO) (10%) (= control) or 10–100 µM of either SNX-ab, Onalespib, PU-H71 or SNX-0723 dissolved in DMSO (10%) for 10 min to assess binding specificity. The incubation solutions were removed and the sections were dipped in tris.HCl 50 mM pH 7.4 + 0.3% BSA at 4 °C. The slices were again air-dried and subsequently incubated with [^11^C]SNX-ab (74 kBq/mL in 200–300 µL tris.HCl 50 mM pH 7.4 + 0.3% BSA) for 10 min at room temperature. Additionally, some slices were co-incubated with [^11^C]SNX-ab (74 kBq/mL in 200–300 µL Tris.HCl 50 mM pH 7.4 + 0.3% BSA) supplemented with DMSO (10%) (= control) or 10–100 µM of either SNX-ab, Onalespib, PU-H71 or SNX-0723 dissolved in DMSO (10%) for 10 min. After incubations, the slices were washed twice for 5 min in Tris.HCl 50 mM pH 7.4 + 0.3% BSA at 4 °C with a final dip in water at 4 °C. In a follow-up experiment, 6.5% EtOH was added to the washing steps to reduce possible non-specific binding to the glass microscope plates. The air-dried slices were exposed to a phosphor storage screen (super-resolution screen; Perkin Elmer, Waltham, MA) overnight. The autoradiograms were obtained by reading the screens using a Cyclone Plus system (Perkin Elmer).

Autoradiography images were analysed using Optiquant software (Perkin Elmer) and results are expressed as digital light units per square mm (DLU/mm^2^). Percentage block versus control was calculated as (1 − (average DLU/mm^2^ in the presence of 100 µM blocker))/(average DLU/mm^2^ tracer only) × 100% on 3–4 tissue sections within the same experiment and expressed as mean ± SD.

### In vitro cell binding studies

U87 cells were seeded at 125,000 cells/well density in the 6-well plates and incubated at 37 °C for 48 h in the presence of 5% CO_2_. Cells were gently washed once with PBS prior to the incubation with 200 µM of either SNX-ab, Onalespib, PU-H71 or SNX-0723 blocking agents dissolved in the cell culture medium (total 1% DMSO) for 60 min at 37 °C and 5% CO_2_ to assess binding specificity. Cell culture medium with 1% DMSO was used as a baseline control. All conditions were tested in triplicates. The cell culture media was removed and the cells were gently washed once with PBS prior to the incubation with 250 kBq/mL of [^11^C]SNX-ab in the cell culture medium (total 1% ethanol) for 30 min at 37 °C. After incubation, cellular uptake was terminated by washing the cells three times with 1 mL of ice-cold PBS. Surface-bound tracer was removed by two consecutive incubations with 1.5 mL of glycine–HCl (50 mM, pH 2.8) for 5 min at room temperature. Cells were washed with 1 mL of ice-cold PBS and lysed by adding 400 µL of the lysis buffer (reagent A100, Chemometec, Allerod, Denmark) and gently mixing by pipetting. The lysis buffer was collected and quenched with 400 µL of the neutralization buffer (reagent B, Chemometec, Allerod, Denmark) by first washing the wells and then adding the neutralization buffer to the lysed fraction. The PBS wash, glycine–HCl washes and lysed fractions were collected separately and the radioactivity in each fraction was counted with an automated gamma counter as described above. The number of cells per well was counted using an automated counting device with nucleocasettes (NucleoCounter^®^ NC-100™, Chemometec). Results were expressed as percentage of the applied radioactivity bound to 1 × 10^6^ cells normalized to the baseline control conditions and plotted with Graphpad Prism 8.4.0 (Graphpad Software) as a mean ± SD.

### *Ex vivo* plasma radio metabolite studies

Healthy C57BL/6 mice were anesthetized using 2.5% isoflurane in O_2_ at a flow rate of 1 L/min and intravenously (i.v.) injected with ~ 5 MBq of [^11^C]SNX-ab via a tail vein. The mice were subsequently sacrificed by decapitation at 10 min post tracer injection (n = 3) and the blood was collected in K_2_EDTA-containing tubes (BD vacutainer, BD, Franklin Lakes, NJ, USA) and stored on ice. The plasma was separated by centrifugation of the blood for 5 min at 2330 × *g.* The isolated plasma was weighed, counted in a gamma counter and spiked with authentic reference compound (10 µL of 1 mg/mL solution in DMSO). The plasma samples were analysed by RP-HPLC on a Chromolith RP-C_18_ column (3 mm × 100 mm, Merck, Darmstadt, Germany) eluted with gradient mixtures of CH_3_CN (A) and NaOAc 0.05 M pH 5.5 (B) (Additional file 1: Table S1). After passing through an in-line UV detector at 254 nm coupled to a 3-inch NaI(Tl) scintillation detector connected to a single channel analyser, the HPLC eluent was collected in 1 mL fractions of which the radioactivity was measured in an automated gamma counter as described above.

### *Ex vivo* biodistribution studies

Healthy C57BL/6 mice were anesthetized using 2.5% isoflurane in O_2_ at a flow rate of 1 L/min and *i.v.* injected with ~ 5.5 MBq of [^11^C]SNX via a tail vein. The mice were subsequently sacrificed by decapitation at 10 or 60 min post tracer injection (n = 3 per time point). All organs of interest, including blood, were collected in tared tubes and weighed. The radioactivity in each organ was counted in an automated gamma counter as described above. For the calculation of the total radioactivity in blood, muscle and bone, the masses were assumed to be respectively 7%, 40% and 12% of the total body mass (Burns et al. 2007; Horti et al. 2006; Vermeulen et al. 2019). The plasma was separated from the blood cell fraction by centrifugation of the blood for 5 min at 2330 × *g*. The bone marrow was isolated from bone (femur) by several washing and centrifugation (2330 × *g*) steps using PBS (Liu and Quan 2015; Lwin et al. 2016) (Liu and Quan 2015). Data were expressed as percentage of injected dose (%ID) and standardized uptake value (SUV) and plotted with Graphpad Prism 8.4.0 (Graphpad Software) as mean ± SD.%ID was calculated as (counts per min (cpm) in organ/total cpm recovered) × 100%. SUV was calculated as (radioactivity in cpm in organ/weight of organ in g)/(total cpm recovered/total body weight in g).

### In vivo µPET imaging studies

µPET imaging studies were performed on healthy C57BL/6 mice. The mice were anesthetized using 2.5% isoflurane in O_2_ at a flow rate of 1 L/min before the start of the scan and kept under anesthesia during the entire scan period. Immediately after i.v. injection with ~ 4 MBq of [^11^C]SNX-ab via a tail vein, the mice were scanned dynamically for 90 min using Molecubes (β-CUBE), followed by a CT scan using Molecubes (X-CUBE), allowing full body field of view PET/CT imaging. To assess Hsp90-specific binding, mice (n = 2 per pre-treatment) were injected intraperitoneally (i.p.) 20 min before tracer injection with either 100–200 µL vehicle or SNX-ab (1 mg/kg) dissolved in an aqueous solution of 5% DMSO and 95% (2-hydroxypropyl)-β-cyclodextrin (40%) in H_2_O. Pre-treatment solutions were sterile filtered through a 0.22 µm membrane filter (Millex-GV, Millipore).

PET data were histogrammed into 17 frames (4 × 15 s, 1 × 38 s, 3 × 60 s, 1 × 180 s, 1 × 450 s, 7 × 600 s) and reconstructed using 30 iterations of the manufacturer’s MLEM algorithm with corrections for randoms, scatter, attenuation and decay into a 192 × 192 image matrix containing 0.4 mm voxels. CT data were reconstructed using a regularized statistical (iterative) image reconstruction algorithm using non-negative least squares, using an isotropic 200 µm voxel size and scaled to Hounsfield Units (HUs) after calibration against a standard air/water phantom. A manual region of interest (ROI’s) was drawn over the whole brain using PMOD software (v3.3, PMOD Technologies, Zürich, Switzerland) after normalizing for injected dose and bodyweight to generate tissue time-activity curves (TACs) scaled to Standard Uptake Value (SUV). TACs were plotted as SUV mean ± SD using Graphpad Prism 8.4.0 (Graphpad Software).

## Results

### Radiosynthesis

**Fig. 2:**
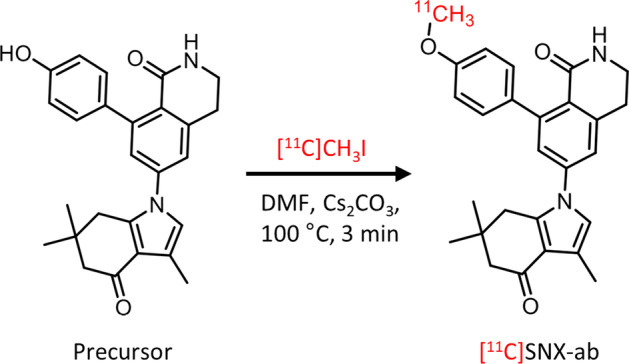
Radiosynthesis of [^11^C]SNX-ab

[^11^C]SNX-ab was synthesized by O-alkylation of the phenol precursor (Fig. 2). The compound was obtained with radiochemical yields of 45 ± 7% relative to total recovered carbon-11 radioactivity on preparative HPLC (Additional file 1: Fig. S12). The QC chromatogram (Additional file 1: Fig. S13) of [^11^C]SNX-ab spiked with authentic reference compound SNX-ab verified the identity of the compound with a radiochemical purity of > 98% and a molar activity of 60 ± 13 GBq/µmol at the end of synthesis (EOS) (n = 3).

### In vitro autoradiography

**Fig. 3:**
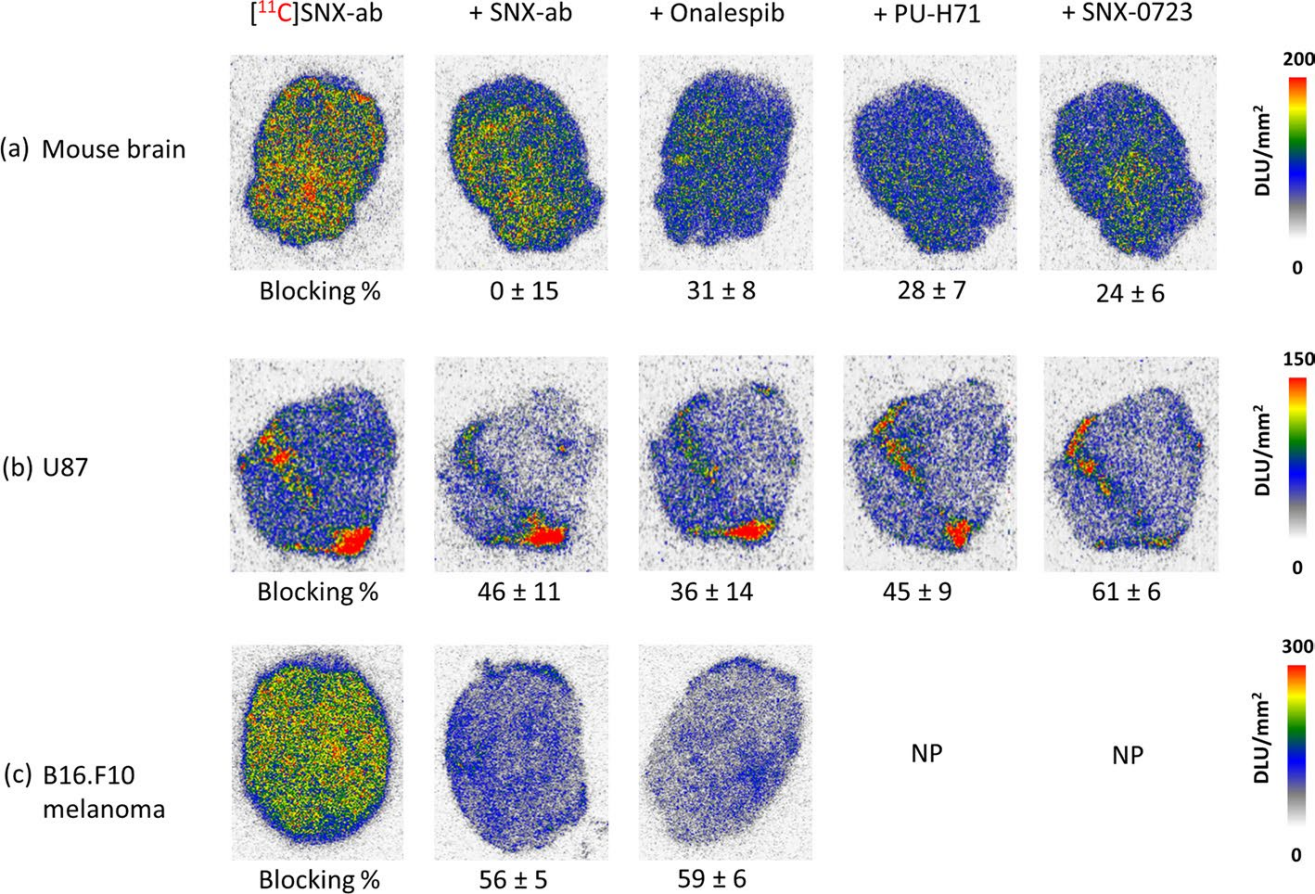
In vitro autoradiography. Slices were incubated with [^11^C]SNX-ab (74 kBq/mL). Binding specificity was assessed by pre-incubation with homologous (SNX-ab) and heterologous (Onalespib, PU-H71, SNX-0723) inhibitors. **a** Mouse brain slices (n = 3), blocking concentrations of 100 µM. **b** U87 slices (n = 3), blocking concentrations of 100 µM. **c** B16.F10 melanoma slices (n = 4), blocking concentrations of 10 µM. Intensity is depicted as DLU/mm^2^. Blocking% was calculated as (1 − (average DLU/mm^2^ in tissue slice in the presence of blocking agent))/(average DLU/mm^2^ in tissue slice tracer only)*100%. Data are presented as mean ± SD. *NP* Not performed

In vitro autoradiography experiments showed [^11^C]SNX-ab binding to healthy mouse brain slices (Figs. 2 and 3a). Binding specificity was assessed by blocking studies using the authentic reference compound (SNX-ab), homologous (SNX-0723) and heterologous (Onalespib and PU-H71) inhibitors (Table 1). Tracer binding was not significantly reduced upon pre-incubation of the slices with the Hsp90 blocking agents, indicated by the low blocking percentages (Figs. 2 and 3a). Additionally, [^11^C]SNX-ab binding to two different tumour sections, U87 glioblastoma and B16.F10 melanoma tissue, was evaluated (Fig. 3b and c). The local high intensity spots on U87 spots were attributed to artefacts resulting from the double folding at the edges of the tissue slice. Similarly, the binding of [^11^C]SNX-ab was only moderately reduced for all blocking agents. Co-incubating the Hsp90 inhibitors together with [^11^C]SNX-ab or increasing their concentrations for pre-incubation from 10 µM up to a relatively high concentration of 100 µM had no significant effect on blocking percentages (Additional file 1: Fig. S14).

### In vitro cell binding studies

**Fig. 4:**
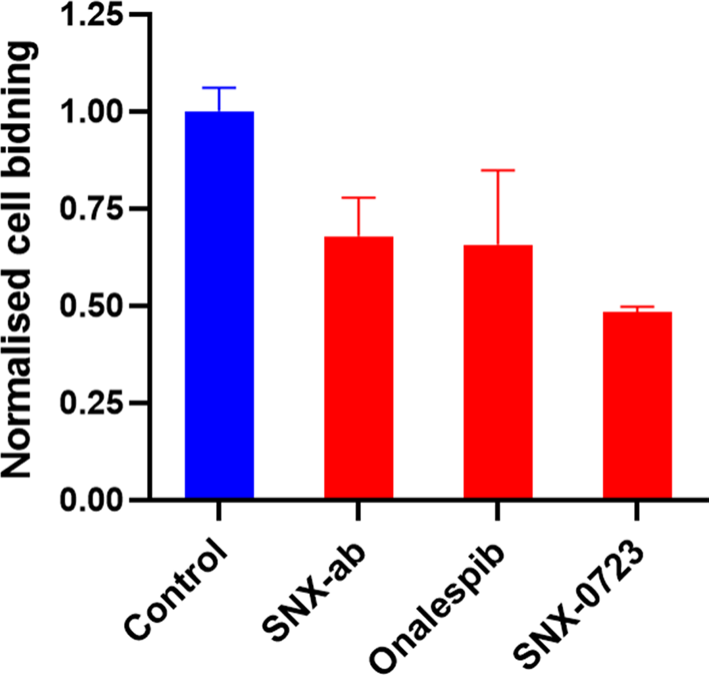
Cell binding study. U87 cells were incubated with [^11^C]SNX-ab (250 kBq/mL). Binding specificity was assessed by pre-incubation with homologous (SNX-ab, SNX-0723) and heterologous (Onalespib) inhibitors (100 µM) at 37 °C (n = 3). Values are expressed as percentage of the applied radioactivity bound to 1 × 10^6^ cells normalized to the value for control cells. Data are expressed as mean ± SD

The binding of [^11^C]SNX-ab to Hsp90 was evaluated by performing a cell binding study on live U87 cells showing relatively low cellular uptake (Additional file 1: Table S2). Binding specificity was evaluated by coincubation with structurally-related (SNX-ab, SNX-0723) and non-related (Onalespib) Hsp90 inhibitors. In agreement with the autoradiography results, pre-incubation of the U87 cells with the above-mentioned Hsp90 inhibitors only reduced intracellular tracer binding to a limited extent (30–50%) (Fig. 4) and had no effect on the membrane-bound radioligand (Additional file 1: Table S2).

### *Ex vivo* plasma metabolite and biodistribution study

The biodistribution of [^11^C]SNX-ab was evaluated in healthy C57BL/6 mice at 10 and 60 min post tracer injection (n = 3 per time point). [^11^C]SNX-ab was predominantly cleared from the plasma by the hepatobiliary system (%ID > 35 at 10 min post tracer injection) combined with partial renal clearance (%ID > 16 at 10 min post tracer injection), indicated by decreasing %ID values in time (Additional file 1: Table S3). A plasma radio-metabolite study conducted on healthy C57BL/6 mice demonstrated the presence of a minimal fraction of polar radio-metabolites as 91 ± 5% of the recovered radioactivity corresponded to intact tracer compound at 10 min post tracer injection (Additional file 1: Fig. S15).

**Fig. 5:**
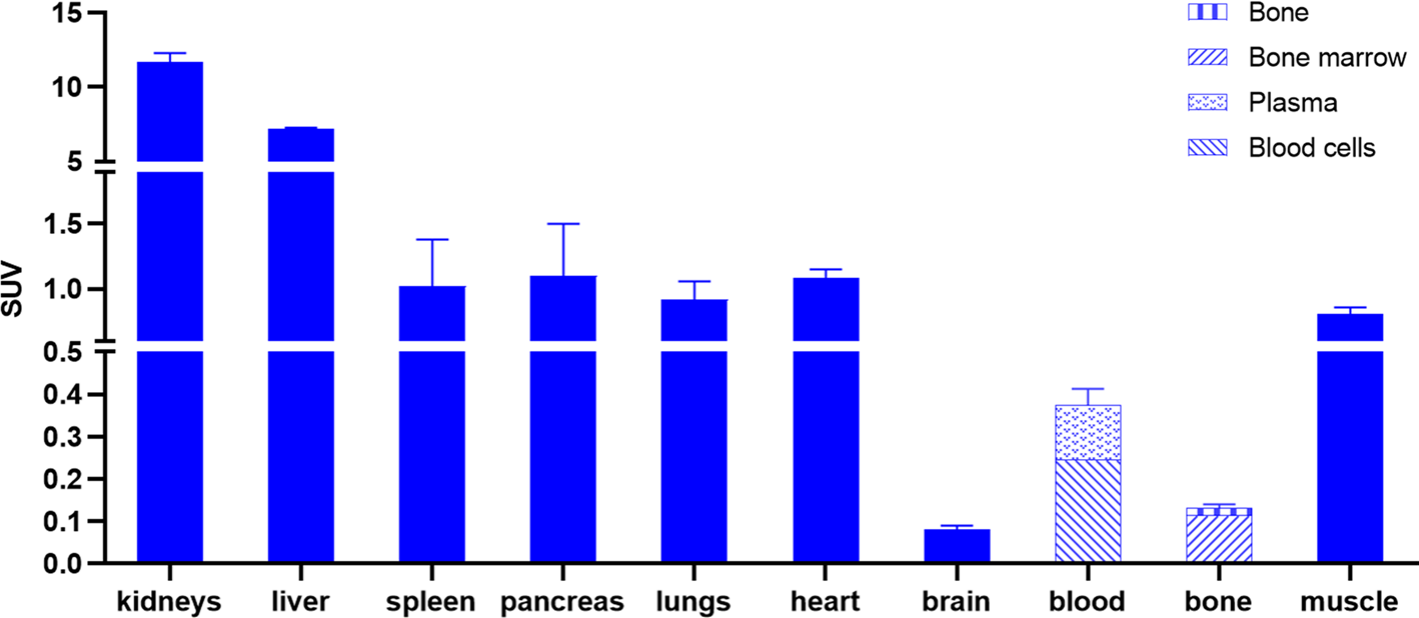
Biodistribution data of [^11^C]SNX-ab in C57BL/6 mice at 10 post tracer injection. Data are expressed as mean ± SD

Organ SUV values indicated that [^11^C]SNX-ab is particularly retained in the kidneys and liver (Fig. 5). [^11^C]SNX-ab showed lower uptake values in spleen, lungs, pancreas, heart and muscle. SUV values in blood and bone were slightly lower, of which binding was predominantly attributed to the blood cell fraction and bone marrow respectively (Additional file 1: Table S4, Fig. 5). Only limited brain uptake of the tracer was observed (SUV < 0.1 at 10 and 60 min post tracer injection).

### In vivo µPET study

**Fig. 6:**
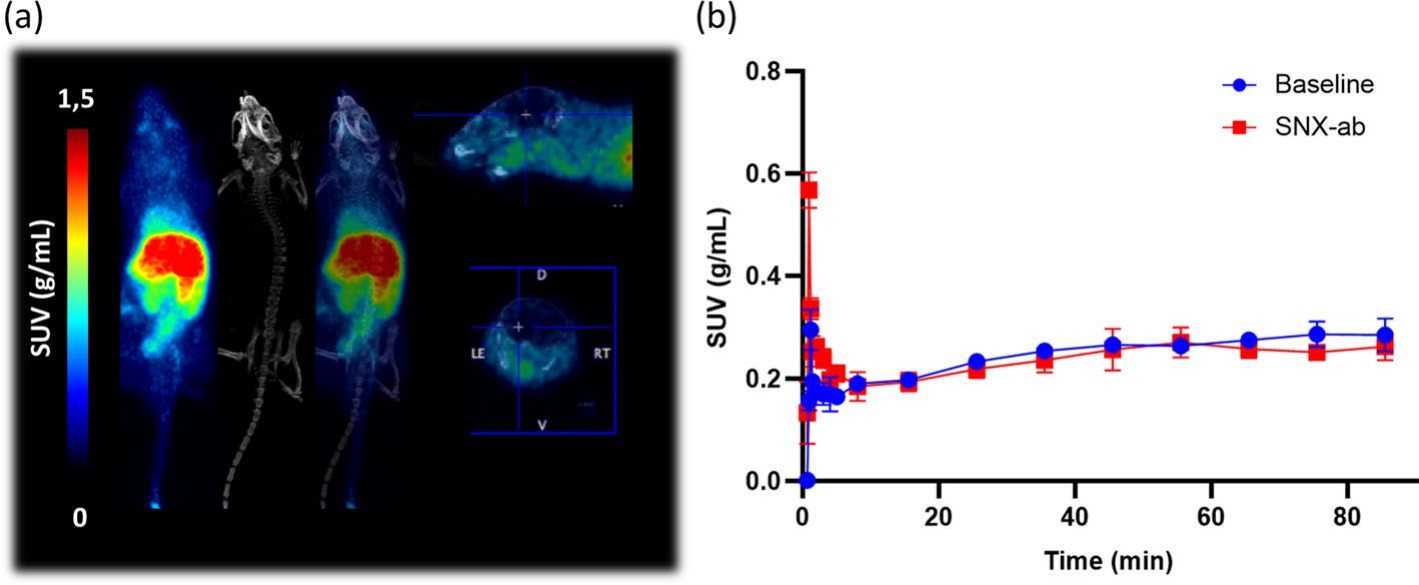
µPET study on healthy C57BL/6 mice. **a** Whole body maximum intensity projection image of 90-min dynamic baseline scan after i.v. injection of [^11^C]SNX-ab (~ 4 MBq, n = 2). **b** Averaged brain SUV TAC’s of µPET study with [^11^C]SNX-ab, mice were pre-treated with an i.p. injection of vehicle (blue) or SNX-ab (1 mg/kg) (red) 20 min before tracer injection. Data are expressed as mean ± SD

A proof-of-concept in vivo dynamic µPET study was performed in healthy C57BL/6 mice. The maximum intensity projection (MIP) PET/CT overlay images (Fig. 6a) revealed no in vivo brain uptake of [^11^C]SNX-ab over the 90-min dynamic scan period. The tracer predominantly accumulated in abdominal regions (Fig. 6a) and showed mainly hepatobiliary clearance consistent with the *ex vivo* biodistribution study (Additional file 1: Table S3). In vivo saturable binding of the tracer was evaluated by pre-treatment of the mice with SNX-ab (1 mg/kg) via i.p. injection 20 min before tracer injection. The quantified brain SUV TAC’s indicated almost identical limited brain uptake for both conditions (Fig. 6b).

## Discussion

Over 400 client proteins are known to be processed by the Hsp90 chaperone machinery. As a result, Hsp90 is one of the most abundant proteins as it accounts for 1–2% of the total cellular protein count in peripheral cells (Hoter et al. 2018). Despite its ubiquitous expression and essential function in the body, Hsp90 inhibition has emerged as an attractive strategy for cancer treatment by enabling the inactivation of many of the multiple proteins that conspire in tumourigenesis (Calderwood 2018).

Tumour Hsp90 is reported to be both overexpressed and more sensitive for Hsp90 inhibitors, creating a potential therapeutic window for Hsp90 inhibitor treatment (Lianos et al. 2015; Miyata et al. 2013). However, clinical evaluation of multiple Hsp90 inhibitors showed adverse effects, possibly caused by both a lack of specificity (Neckers et al. 2018) and target-mediated drug disposition (TMDD), leading to dose dependent non-linear kinetics of the Hsp90 inhibitors (Badolo et al. 2020; Yamazaki et al. 2013). This indicates that targeting of Hsp90 is a challenging fine balance between the intended result of eradicating malignant cells and the menace to healthy tissues (Calderwood 2018).

Targeting Hsp90 isoforms has emerged as an alternative strategy to address the adverse effects of pan-Hsp90 inhibitors, by reducing the number of client proteins affected and retaining their functions (Li et al. 2020). The development of a novel benzolactam series of potent selective (> 1000 fold) Hsp90α/β inhibitor compounds with less cellular toxicity was achieved by Ernst et al. (2014b). As such, the improved tolerability profiles may allow the use of HSP90α/β selective inhibitors as therapeutics for neurodegenerative diseases, including HD.

In this regard, we selected a Hsp90α/β isoform-selective compound (SNX-ab, Fig. 1) from the series of benzolactam Hsp90 inhibitors described by Ernst et al. (2014b) with favorable properties regarding BBB permeability (Table 1) suitable for straight-forward radiolabeling with carbon-11. We successfully developed a radiolabeling method with high radiochemical yields (> 40%, Additional file 1: Fig. S12) to obtain the [^11^C]SNX-ab isoform-selective PET brain tracer (Fig. 2), which could potentially be used to visualize and quantify Hsp90α/β expression levels in vivo in health and disease and hence support the development of new isoform-specific Hsp90 therapeutics. The in vivo Hsp90α/β PET data may contribute to the understanding of the specific functions of the unique isoforms, which, together with the possibility to quantify Hsp90α/β occupancy as a function of the dose of the Hsp90 therapeutic under investigation, may enhance Hsp90 therapeutic development.

[^11^C]SNX-ab was evaluated in in vitro autoradiography blocking experiments. The brain-permeable orally active compound SNX-0723 (Fig. 1, Table 1), which possesses a modest level of HSP90α/β isoform selectivity, was included as homologous inhibitor. SNX-0723 is described to show significant brain exposure resulting in induction of brain Hsp70 in vivo and prevent α-synuclein oligomerization resulting in rescued α-synuclein cytotoxicity in vitro (Putcha et al. 2010). Additionally, SNX-0723 successfully displaced binding of our previously developed PET probe [^11^C]YC-72-AB85 to brain Hsp90 in both in vitro and in vivo experiments (Vermeulen et al. 2021). Onalespib and the epichaperome inhibitor PU-H71 (Rodina et al. 2016; Taldone et al. 2020), representatives of the resorcinol and purine bearing class respectively are both Hsp90 inhibitors that have been evaluated in clinical trials, and were chosen as non-structurally related competitive Hsp90 inhibitors (Fig. 1, Table 1). Although SNX-ab was described as a highly potent and selective Hsp90α/β inhibitor (Ernst et al. 2014b), our in vitro autoradiography results indicated that binding of [^11^C]SNX-ab to both rodent brain and tumour (U87 and B16.F10 melanoma) tissue slices was not entirely Hsp90-specific, as blocking with relatively high concentrations of the authentic reference compound (SNX-ab), homologous inhibitor (SNX-0723) and heterologous pan-selective inhibitors (Onalespib, PU-H71) did result in only limited reduction of tracer binding. Of note, efforts were made in optimizing washing steps to reduce potential non-specific binding to the glass plates on which sections were mounted, however, with no favourable results. Blocking percentages were slightly higher for tumour tissue as compared to brain tissue, but still only in the range of 30–60%, which is considerably lower as compared to autoradiography specificity studies with our previously developed probe [^11^C]YC-72-AB85 (80–100%) (Vermeulen et al. 2021).

The Hsp90 expressing human primary glioblastoma cell line U87 (Cruickshanks et al. 2010) was used in a [^11^C]SNX-ab cell binding study. In agreement with autoradiography results to U87 tumour tissue slices, both homologous (SNX-ab, SNX-0723) and heterologous (Onalespib) inhibitors showed no efficient blocking of intra- and extracellular tracer binding, although these blocking agents were able to reduce tracer binding up to 95% in U87 cell binding experiments using our previously developed Hsp90 PET probes (Cools et al. 2022).

*Ex vivo* biodistribution studies in healthy mice indicated that the tracer showed predominantly hepatobiliary clearance, which was similar for our previously developed Hsp90 PET probes, [^11^C]NMS-E973 (Vermeulen et al. 2019) and [^11^C]YC-72-AB85 (Vermeulen et al. 2021) (Fig. 1), and to be expected as the tracer is highly lipophilic (Table 1). Compared to [^11^C]NMS-E973 and [^11^C]YC-72-AB85, [^11^C]SNX-ab showed significantly higher accumulation in liver (SUV_10 min, liver_ = 7.2 for [^11^C]SNX-ab versus SUV_10 min, liver_ = 4.4 for [^11^C]NMS-E973 and 2.9 for [^11^C]YC-72-AB85) and especially in kidney (SUV_10 min, kidney_ = 11.7 for [^11^C]SNX-ab versus SUV_10 min, kidney_ = 4.4 for [^11^C]NMS-E973 and 2.3 for [^11^C]YC-72-AB85). Plasma analysis at 10 min post tracer injection proved slow tracer metabolism as more than 90% of the radioactivity levels in plasma still corresponded to the intact tracer compound. Surprisingly, no significant brain uptake of [^11^C]SNX-ab was observed. Since autoradiography and cell binding studies showed that binding of [^11^C]SNX-ab was not fully Hsp90-specific, no further extensive biodistribution studies were performed.

Although the physicochemical parameters of SNX-ab (Table 1) are rather favourable for free diffusion across the BBB (Pike 2009), the in vivo dynamic µPET studies in healthy mice confirmed limited brain exposure of [^11^C]SNX-ab. Possibly, the compound is a substrate for P-glycoprotein (P-gp) efflux transporter. These findings confirm the complexity of the biological logistics of drug delivery to the CNS, which challenges the development of CNS therapeutics (Dinunzio et al. 2008). Successful in vitro studies concerning therapeutic CNS molecules do not guarantee promising in vivo results often due to their inability to cross the BBB resulting in too low brain exposure. An in vivo pre-treatment study using cyclosporin, a potent P-gp inhibitor, could be implemented to evaluate the influence on brain uptake of [^11^C]SNX-ab and potentially support our findings regarding BBB permeability (Liow JS et al. 2007). Additionally, a non-human primate PET study could be included as species differences in both P-gp expression and activity could result in inaccurate estimation of BBB penetration (Verscheijden et al. 2021), but this has not been performed in our study due to insufficient Hsp90-specific binding of the tracer in in vitro experiments which already disqualified the probe for its intended use.

Our results suggest that despite the promising reported characteristics of SNX-ab (Ernst et al. 2014b), [^11^C]SNX-ab could not be used for quantification of Hsp90α/β expression levels in tumour and brain, considering the insufficient specific Hsp90 binding of [^11^C]SNX-ab observed in our in vitro experiments and the limited brain exposure shown by our *ex vivo*/in vivo studies. The latter is often a bottleneck in the development of CNS therapeutic and PET probes. Onalespib (Canella et al. 2017) and NMS-E973 (Brasca et al. 2013; Sun et al. 2018) for example were initially also reported to cross the BBB. Although brain uptake might have been sufficient to induce a pharmacological effect, our own findings showed consistently that both compounds did not show sufficient brain uptake for PET imaging (Vermeulen et al. 2021; Vermeulen et al. 2019), which supports the relevance of PET as a tool to investigate in vivo behaviour of specific probes.

Our recent findings using [^11^C]YC-72-AB85 demonstrated saturable Hsp90 binding pools in tumour, blood, blood-rich organs and brain (Vermeulen et al. 2021). These saturable binding pools are intriguing and raise new questions regarding the relative presence of specific isoforms in the observed high affinity saturable binding pools. PET using a subtype-specific Hsp90 probe could potentially show completely different pharmacokinetic behaviour, which could provide new insights in this matter. Additionally, novel approaches for Hsp90 targeted therapy intend to avoid harmful side effects associated with pan-inhibition by using non-traditional modulation of Hsp90, including C-terminal inhibition or isoform-selective inhibition. The research group of Blagg has recently made a major contribution to this research area by developing several new isoform-selective inhibitors (Chaudhury et al. 2021; Crowley et al. 2017, 2016; Khandelwal et al. 2018, 2017; Mishra et al. 2021a, 2021b, 2017; Muth et al. 2014), including the more challenging development of Hsp90α (Mishra et al. 2021a) and Hsp90β (Chaudhury et al. 2021; Khandelwal et al. 2018; Mishra et al. 2021b) selective compounds. Their Hsp90β-selective inhibitors were claimed to be cytotoxic to cancer cells while not inducing a HSR, thereby overcoming the detriments associated with pan-Hsp90 inhibition (Mishra et al. 2021b). Accordingly, we believe that the development of next-generation isoform-specific Hsp90 PET probes is warranted to support these exciting new results.

## Conclusion

We efficiently radiolabeled and evaluated [^11^C]SNX-ab in vitro and in vivo as an Hsp90α/β isoform selective PET tracer. In vitro autoradiography and cell binding studies indicated that binding of [^11^C]SNX-ab was not fully Hsp90-specific. *Ex vivo* biodistribution studies in healthy mice showed low brain uptake, high tracer uptake in excretion organs and preferential hepatobiliary clearance. In vivo PET imaging using [^11^C]SNX-ab confirmed low brain exposure and tracer accumulation in the abdominal regions due to the hepatobiliary and renal clearance. The results suggest that [^11^C]SNX-ab is not an ideal PET probe for quantification of Hsp90α/β expression levels in tumour and brain due to low Hsp90-specific binding. Given the high added value of Hsp90 isoform subtype specific PET (brain) probes, future research in the development of next-generation Hsp90 isoform-selective tracers is warranted.

## Supplementary Information

**Additional file 1.** Supplementary organic chemistry and biological experiments. Organic synthesis reaction scheme of reference and precursor compound including NMR data of all intermediate and final compounds. Additional biological experimental data, including preparative tracer HPLC, QC tracer HPLC, ARX, cell binding, metabolites and biodistribution.

## Data Availability

All data generated or analyzed during this study are included in this published article and its supplementary information files.

## Abbreviations

Hsp90: Heat shock protein 90
ER: Endoplasmic reticulum
Grp94: 94 KDa glucose-regulated protein
TRAP1: Tumour necrosis factor receptor-associated protein 1
AD: Alzheimer’s disease
PD: Parkinson’s disease
HD: Huntington’s disease
CNS: Central nervous system
HSR: Heat shock response
mHtt: Mutant huntingtin protein
BBB: Blood–brain barrier
PET: Positron emission tomography
EOS: End of synthesis
MIP: Maximum intensity projection
TMDD: Target-mediated drug disposition
P-gp: P-glycoprotein
tPSA: Total polar surface area
LC–MS: Liquid chromatography–mass spectrometry
NMR: Nuclear magnetic resonance
HPLC: High-performance liquid chromatography
ATCC: American type culture collection
DMEM: Dulbecco's modified Eagle's medium
FBS: Fetal bovine serum
DMF: Dimethylformamide
QC: Quality control
DMSO: Dimethylsulfoxide
DLU: Digital light units
i.v.: Intravenously
%ID: Percentage of injected dose
SUV: Standardized uptake value
Cpm: Counts per min
i.p.: Intraperitoneally
ROI: Region of interest
TAC: Time-activity curve
