# Supplementary material for: Radiosynthesis and preclinical evaluation of [11C]SNX-ab as an Hsp90α,β isoform-selective PET probe for in vivo brain and tumour imaging
Source: EJNMMI Radiopharm Chem. 2023 Jan 30;8:2. doi: 10.1186/s41181-023-00189-0 (PMC9886718; doi:10.1186/s41181-023-00189-0)
Supplement: Supplementary file 1 — Additional file 1. Supplementary organic chemistry and biological experiments. Organic synthesis reaction scheme of reference and precursor compound including NMR data of all intermediate and final compounds. Additional biological experimental data, including preparative tracer HPLC, QC tracer HPLC, ARX, cell binding, metabolites and biodistribution. [file 41181_2023_189_MOESM1_ESM.docx]

**Supporting information file 1**

**Chemistry**





**Figure S1** Organic synthesis scheme of reference compound (**4**).

**6,8-difluoro-3,4-dihydroisoquinolin-1(2*H*)-one (2).** To a stirring solution of **1** (1.0 g, 5.95 mmol) in MeSO_3_H (7.0 mL), NaN_3_ (0.541 g, 8.32 mmol) was added at 0 °C (small portions during 10 min). After 30 min, the solution was warmed to room temperature and the reaction was allowed to proceed for an additional 2 h. The reaction mixture was placed in an ice bath and the pH was adjusted to 10 using a 20% NaOH solution. The organic phase was extracted using Et_2_O (3x) and dried over MgSO_4_. The solvent was removed under reduced pressure to give **2** as a white solid (0.436 g, 2.38 mmol,
yield: 40%). ^1^H NMR (300 MHz, DMSO-*d*_6_) δ[ppm]: 8.00 (br s, 1H), 7.18-7.09 (m, 2H), 3.29-3.27 (m, 2H), 2.90 (t, *J* = 6.3 Hz, 2H).

**6-fluoro-8-(4-methoxyphenyl)-3,4-dihydroisoquinolin-1(2*H*)-one (3).** To a stirring solution of **2** (0.081g, 0.44 mmol) in dried THF, 4-methoxyphenylmagnesium bromide (3.49 mL, 0.5 M solution) was added at 0 °C under N_2_ atmosphere. The reaction mixture was then heated to reflux for 24 h and quenched with 10% MeOH in DCM (1 mL). The solvent was removed under reduced pressure and the resultant solid was recrystallized using hot EtOH to give **3** as a white solid (0.056 g, 0.21 mmol, yield: 47%). ^1^H NMR (300 MHz, CD_3_CN) δ[ppm]: 7.22 (d, *J* = 8.7 Hz, 2H), 7.01 (dd, *J* = 8.7 Hz, 1H), 6.95-6.88 (m, 3H), 6.32 (br s, 1H), 3.81 (s, 3H), 3.43 (m, 2H) 2.95 (t, *J* = 6.2 Hz, 2H).

**8-(4-methoxyphenyl)-6-(3,6,6-trimethyl-4-oxo-4,5,6,7-tetrahydro-1*H*-indol-1-yl)-3,4-dihydroisoquinolin-1(2*H*)-one (4) = Reference compound.** To a solution of 3,6,6-trimethyl-6,7-dihydro-1*H*-indol-4(5*H*)-one (0.021 g, 0.118 mmol) in dry DMF, NaH (5.7 mg 0.032 mmol, 60% in mineral oil) was added and stirred at room temperature under N_2_ atmosphere. After 15 min, compound **3** was added (0.032 g, 0.118 mmol) and the reaction mixture was stirred for 24 h at 150 °C. The organic solvent was removed under reduced pressure and the crude product was purified by silica gel column chromatography using heptane/EtOAc (3:7 v/v) as eluent to give **4** as a brown solid (32.1 mg, 0.075 mmol, yield: 63 %). ^1^H NMR (300 MHz, DMSO-*d*_6_) δ[ppm]: 7.94 (s, 1H), 7.38 (d, *J* = 1.6 Hz, 1H), 7.25 (m, 2H) 7.14 (d, *J* = 1.8 Hz, 1H) 6.91 (m, 3H), 3.79 (s, 3H), 3.40 (m, 2H), 2.96 (t, *J* = 5.5 Hz, 2H) 2.75 (s, 2H), 2.26 (s, 2H), 2.21 (s, 2H), 1.00 (s, 6H). ^13^C NMR (75 MHz, DMSO-*d*_6_) δ[ppm]: 194.22, 163.96, 158.92, 144.31, 143.58, 142.14, 139.70, 133.95, 130.22, 127.24, 125.48, 121.98, 121.49, 119.21, 118.85, 113.49, 55.52, 52.50, 36.98, 35.68, 30.34, 28.44, 11.79.

**

**

**Figure S2** Organic synthesis scheme of precursor compound (**9**).

**8-bromo-6-fluoro-3,4-dihydroisoquinolin-1(2*H*)-one (6).** To a stirring solution of **5** (0.5 g, 2.18 mmol) in MeSO_3_H (5.0 mL), NaN_3_ (0.2 g, 3.08 mmol) was added at 0 °C (small portions during 10 min). After 30 min, the solution was warmed to room temperature and the reaction was allowed to proceed for an additional 12 h. The reaction mixture was placed in an ice bath and the pH was adjusted to 10 using a 20% NaOH solution. After extraction of the organic phase with Et_2_O (3x) followed by solvent removal under reduced pressure, the crude product was purified by normal-phase flash column chromatography using CH_3_CN/DCM as eluent (98:2 to 90:10 v/v, 25 min) to give **6** as a brown oil (0.143 g, 0.58 mmol, yield 26.8%). ^1^H NMR (300 MHz, CD_3_COCD_3_) δ[ppm]: 7.40 (dd, *J* = 8.7 Hz, 1H), 7.34 (br s, 1H), 7.17 (dd, *J* = 8.6 Hz, 1H,) 3.46 (m, 2H), 3.02 (t, *J* = 6.3 Hz, 2H).

**8-(4-(benzyloxy)phenyl)-6-fluoro-3,4-dihydroisoquinolin-1(2*H*)-one (7).** A solution of **6** (0.12 g, 0.49 mmol) in EtOH/H_2_O (10:1, 5.0 mL) was heated to reflux and 2-(4-(benzyloxy)phenyl)-4,4,5,5-tetramethyl-1,3,2-dioxaborolane (0.18 g, 0.58 mmol), K_2_CO_3_ (0.1 g, 0.72 mmol) and SiliaCat
DPP-Pd (19.6 mg, 0.25 mmol/g) were added. After 16 h, the reaction mixture was filtered and the collected organic phase was dried under reduced pressure. The crude reaction mixture was purified by reversed-phase flash column chromatography using H_2_O/CH_3_CN as eluent (95:5 to 90:10 v/v, 20 min) to give **7** as a brown oil (30.24 mg, 0.58 mmol, yield: 17.8%). ^1^H NMR (300 MHz, CD_3_COCD_3_) δ[ppm]: 7.51 (m, 2H), 7.37 (m, 3H), 7.22 (m, 2H) 7.06 (dd, *J* = 8.8 Hz, 1H), 6.97 (m, 2H), 6.91 (dd, *J* = 10 Hz, 1H), 5.14 (s, 2H), 3.51 (m, 2H), 3.00 (t, 6.2 Hz, 2H).

**8-(4-(benzyloxy)phenyl)-6-(3,6,6-trimethyl-4-oxo-4,5,6,7-tetrahydro-1H-indol-1-yl)-3,4-dihydroisoquinolin-1(2*H*)-one (8).** To a solution of 3,6,6- trimethyl-6,7-dihydro-1*H*-indol-4(5*H*)-one (15.4 mg, 0.087 mmol) in dry DMF (5.0 mL), compound **7** (30.2 mg, 0.087 mmol) and NaH (7.0 mg, 60 % in oil) were added and stirred under N_2_ atmosphere at 150 °C overnight. The crude product was extracted with DCM/brine and dried over MgSO_4_. The organic phase was removed under reduced pressure and the resultant product was purified by reversed-phase flash column chromatography using H_2_O/CH_3_CN as eluent (95:5 to 90:10 v/v, 20 min) to give **8** as a brown solid (17.3 mg, 0.034 mmol, yield: 39.1%).^1^H NMR (300 MHz, CD_3_CN) δ[ppm]: 7.44 (m, 5H), 7.26 (m, 3H), 7.14 (d, *J* = 2.0 Hz, 1H), 6.98 (m, 2H), 6.75 (s, 1H), 6.41 (br s, 1H), 5.13 (s, 2H), 3.48 (m, 2H), 3.01 (t, *J* = 6.1 Hz, 2H), 2.73 (s, 2H), 2.28 (s, 2H), 2.25 (s, 3H), 1.03 (s, 6H).

**8-(4-hydroxyphenyl)-6-(3,6,6-trimethyl-4-oxo-4,5,6,7-tetrahydro-1*H*-indol-1-yl)-3,4 dihydroisoquinolin-1(2*H*)-one (9) = Precursor compound.** To a solution of **8** (15 mg, 0.030 mmol) in MeOH (10.0 mL), ammonium formate (13.4 mg, 0.21 mmol), 10% Pd/C (6.7 mg) and
10% Pd(OH)_2_/C (6.7 mg) were added. The reaction mixture was heated to reflux and stirred overnight. The mixture was then filtered over Celite and washed with MeOH. The organic solvent was removed under reduced pressure and the crude product was purified by reversed-phase flash column chromatography using H_2_O/CH_3_CN as eluent (95:5 to 90:10 v/v, 20 min) to give **9** as a brown solid (9.53 mg, 0.023 mmol, yield: 76.7%).^1^H NMR (300 MHz, DMSO-*d*_6_) δ[ppm]: 9.42 (br s , 1H), 7.93 (br s, 1H), 7.34 (d, *J* = 1.5 Hz, 1H), 7.12 (m, 3H), 6.92 (s, 1H), 6.72 (m, 2H), 3.38 (m, 2H), 2.95 (t, *J* = 5.3 Hz, 2H), 2.74 (s, 2H), 2.25 (s, 2H), 2.21 (s, 3H), 1.00 (s, 6H). (75 MHz, DMSO-*d*_6_) δ[ppm]: ^13^C NMR (75 MHz, DMSO) δ 194.23, 164.06, 157.13, 144.71, 143.55, 142.15, 139.65, 132.25, 130.22, 127.20, 125.44, 121.72, 121.49, 119.16, 118.81, 114.91, 52.50, 36.97, 35.69, 30.41, 28.44, 11.79.


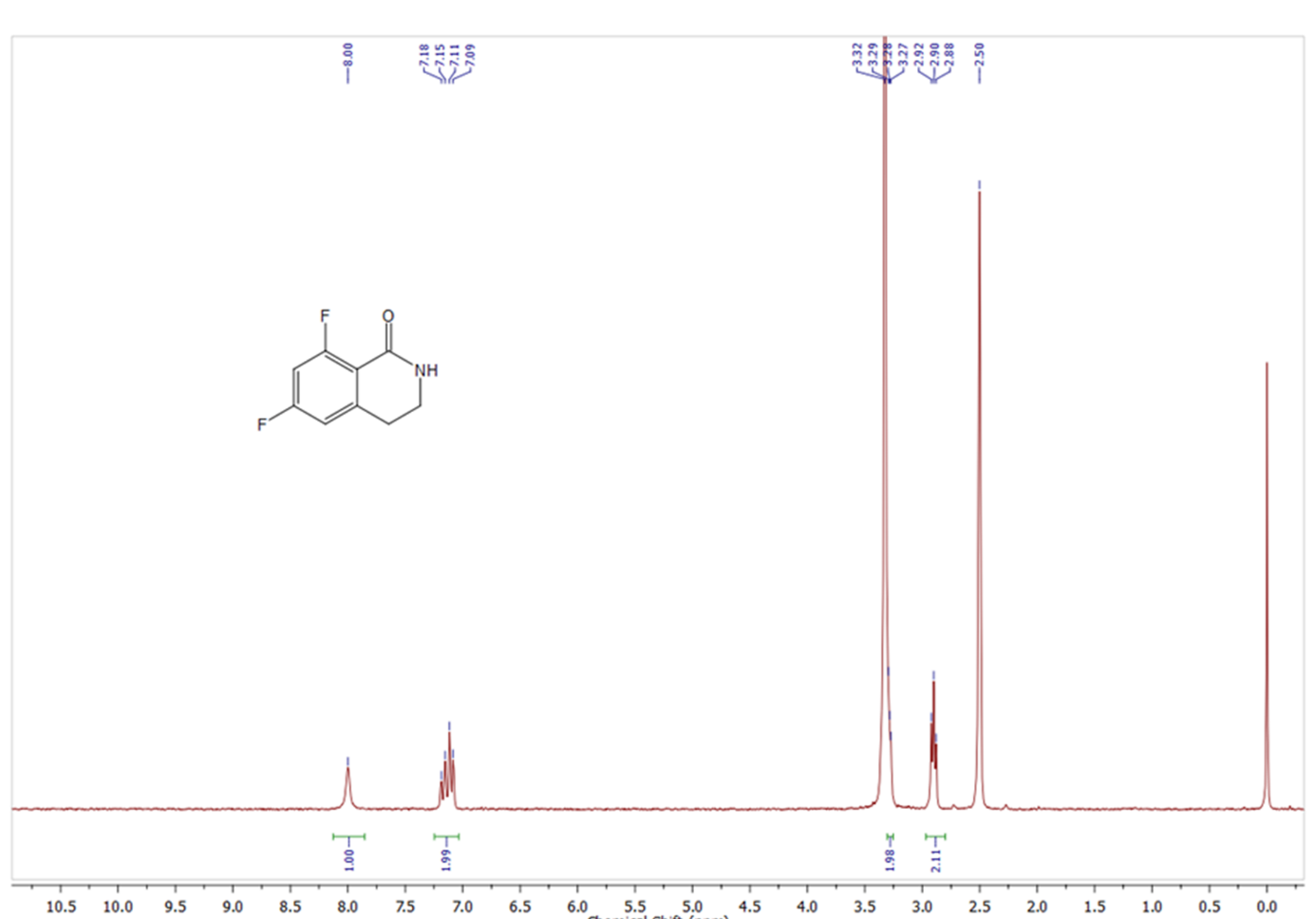


**Figure S3** ^1^H spectrum of **2** (DMSO-*d6*, 300 MHz).

**
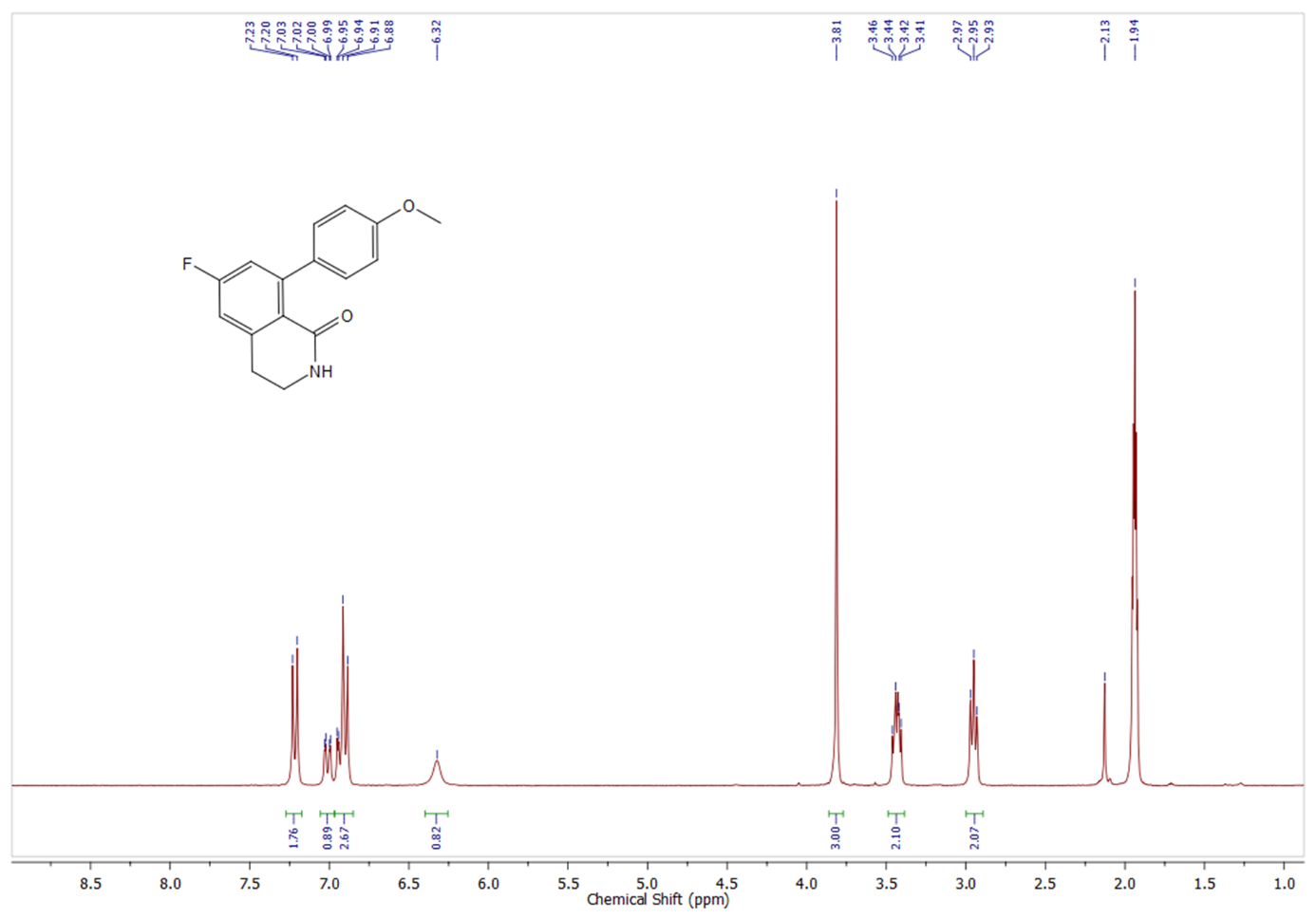
Figure S4** ^1^H spectrum of **3** (CD_3_CN, 300 MHz).

**
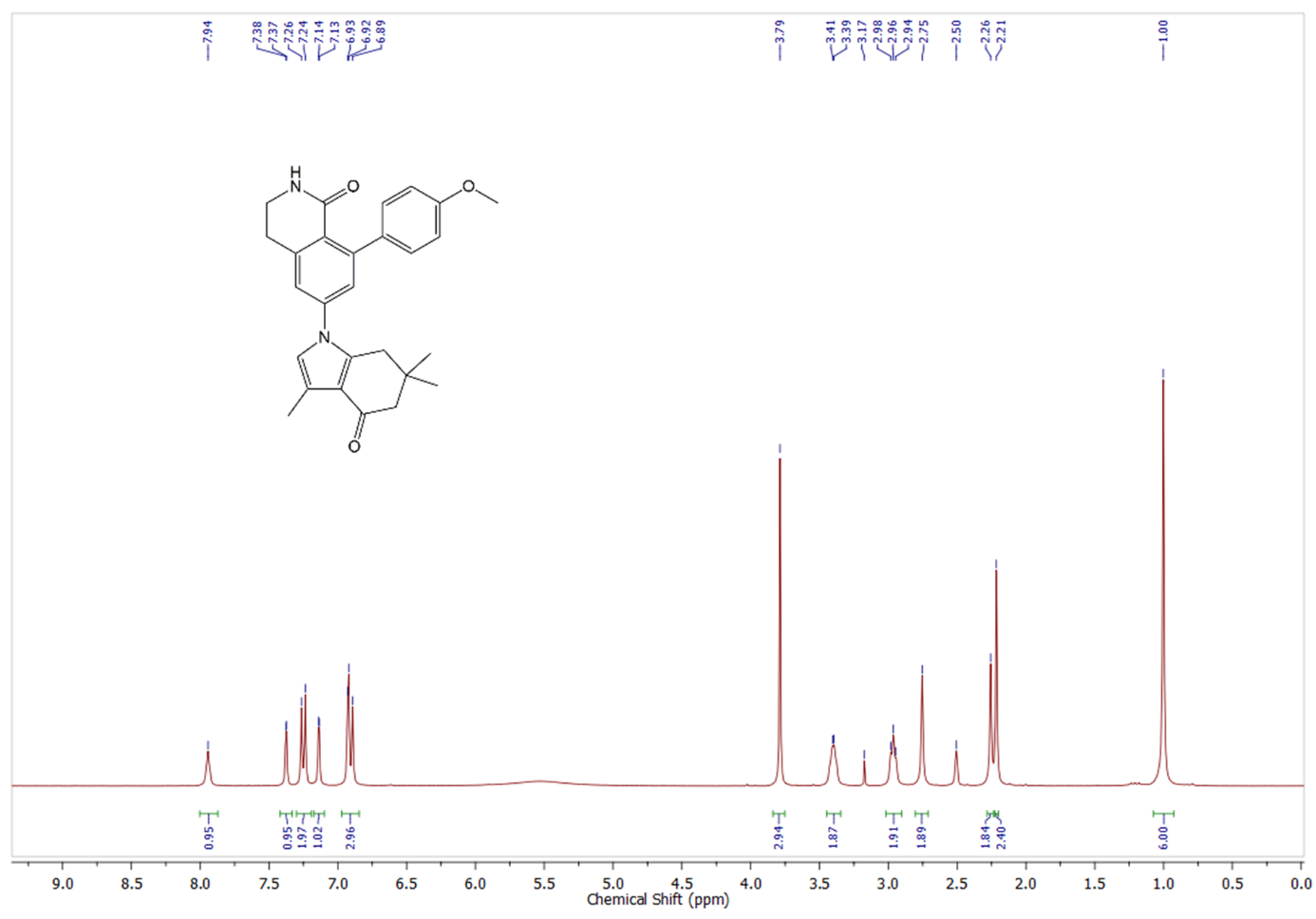
Figure S5** ^1^H spectrum of **4** (DMSO-*d*_6_, 300 MHz).

**
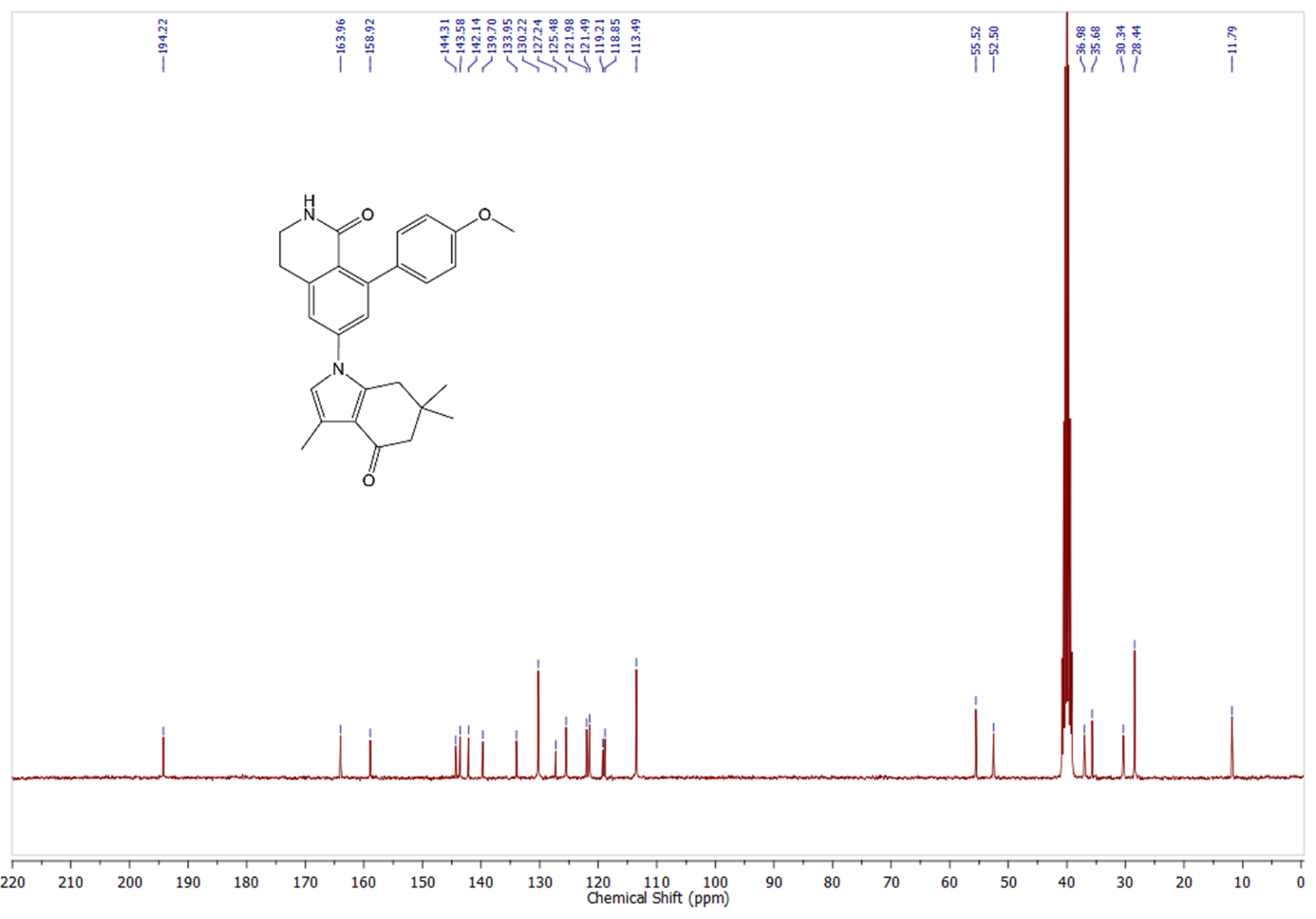
Figure S6** ^13^C spectrum of **4** (DMSO-*d*_6_, 75 MHz).

**
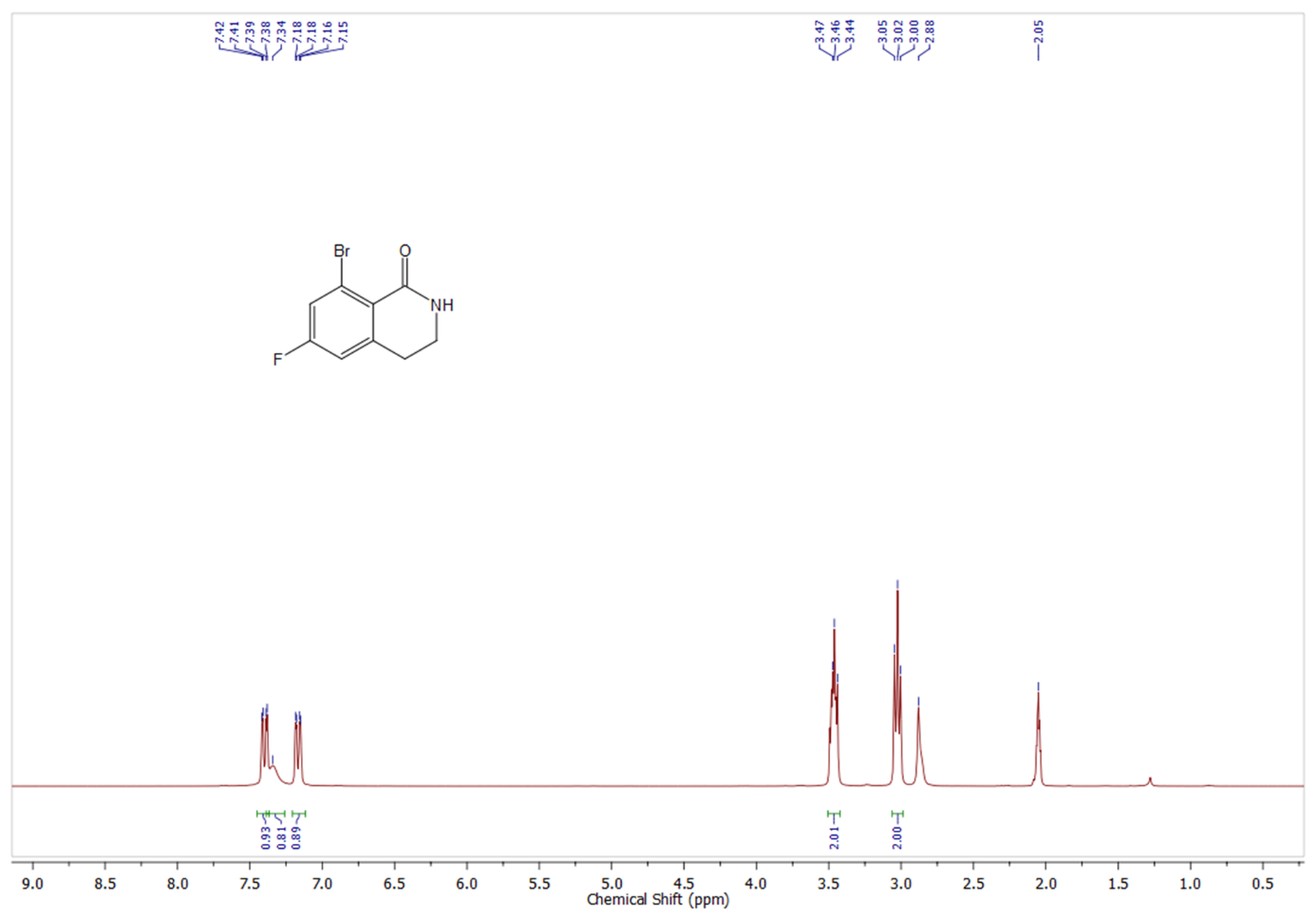
Figure S7** ^1^H spectrum of **6** (CD_3_COCD_3,_ 300 MHz).**
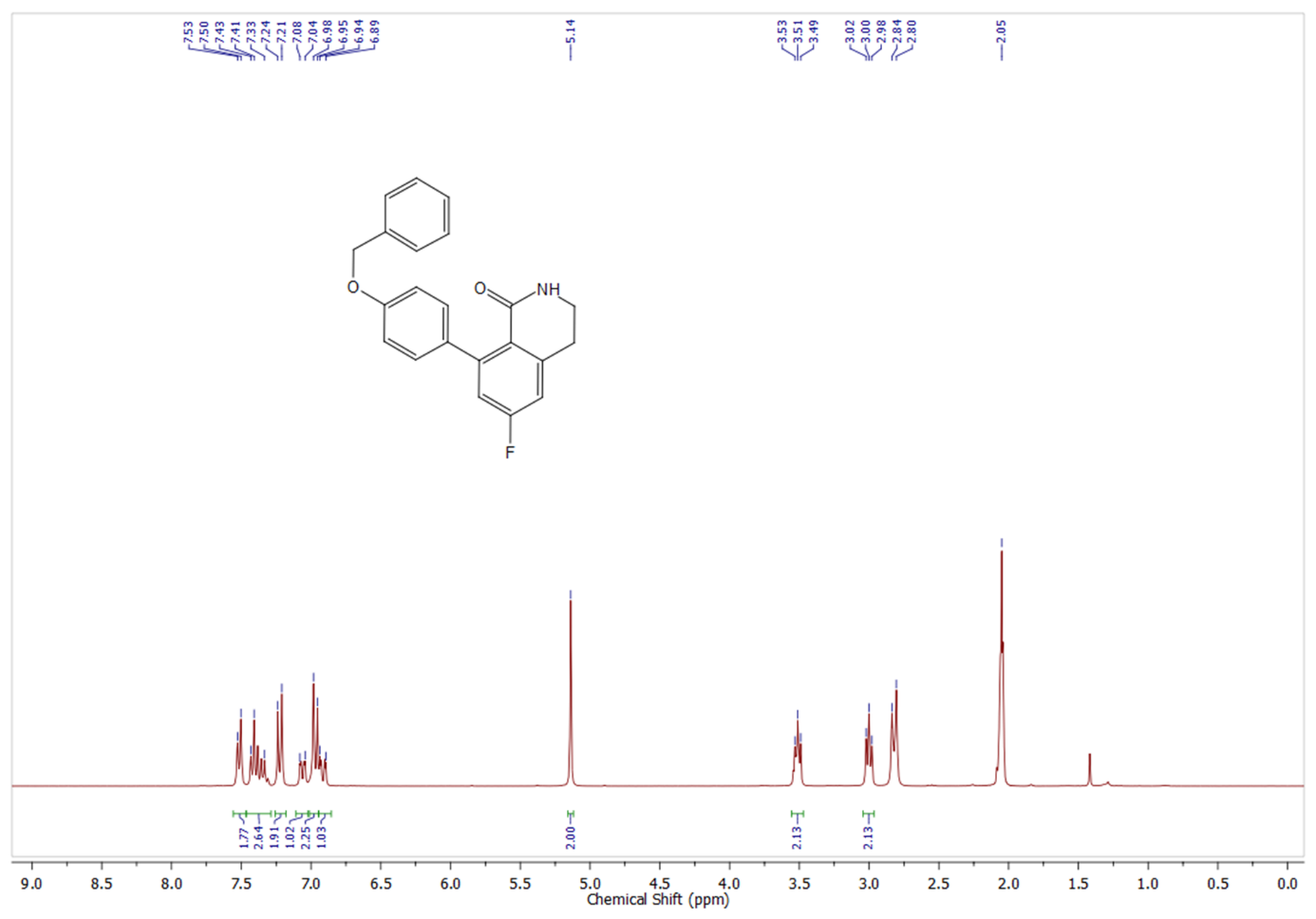
Figure S8** ^1^H spectrum of **7** (CD_3_COCD_3,_ 300 MHz).

**
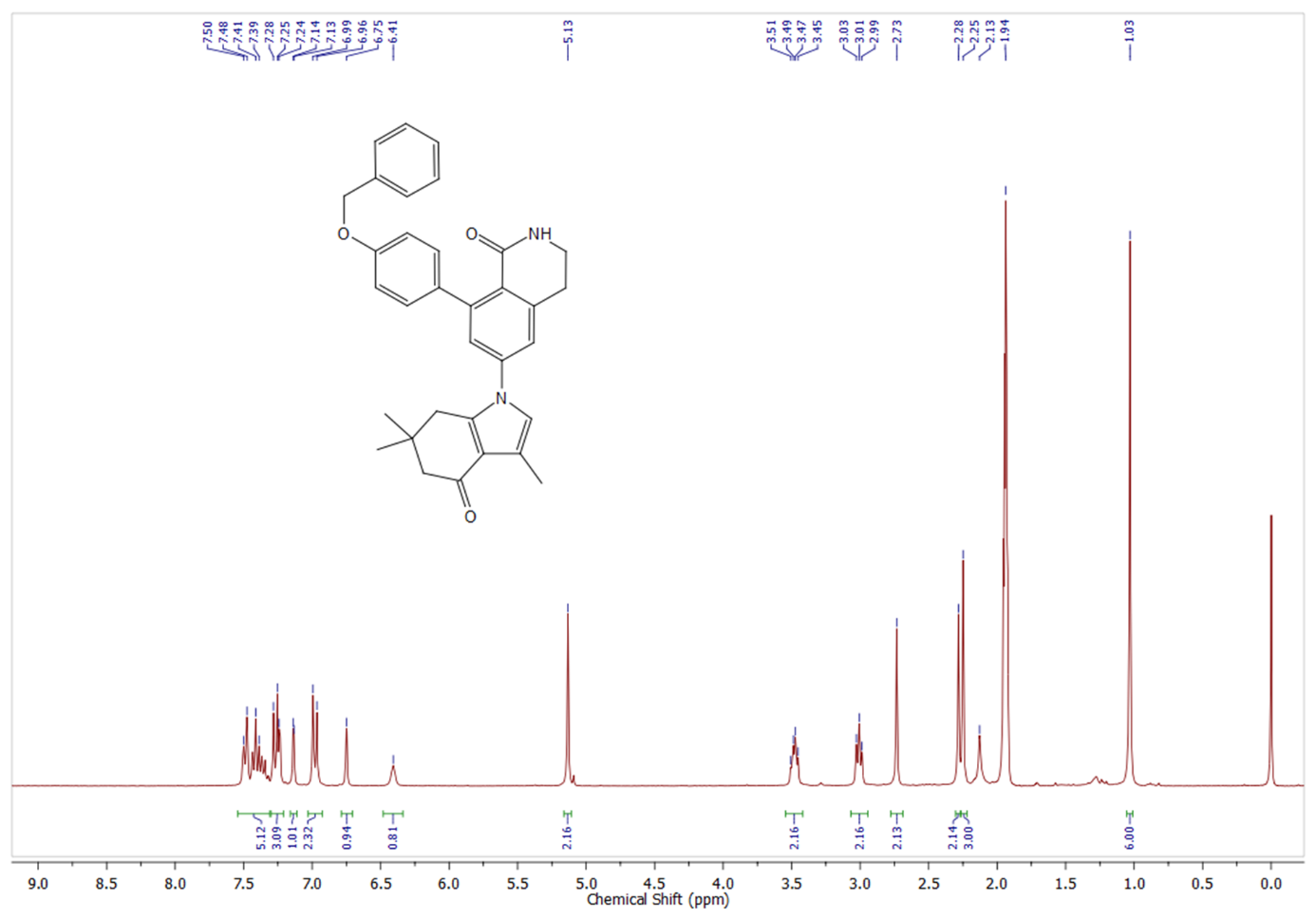
Figure S9** ^1^H spectrum of **8** (CD_3_CN_,_ 300 MHz).

**
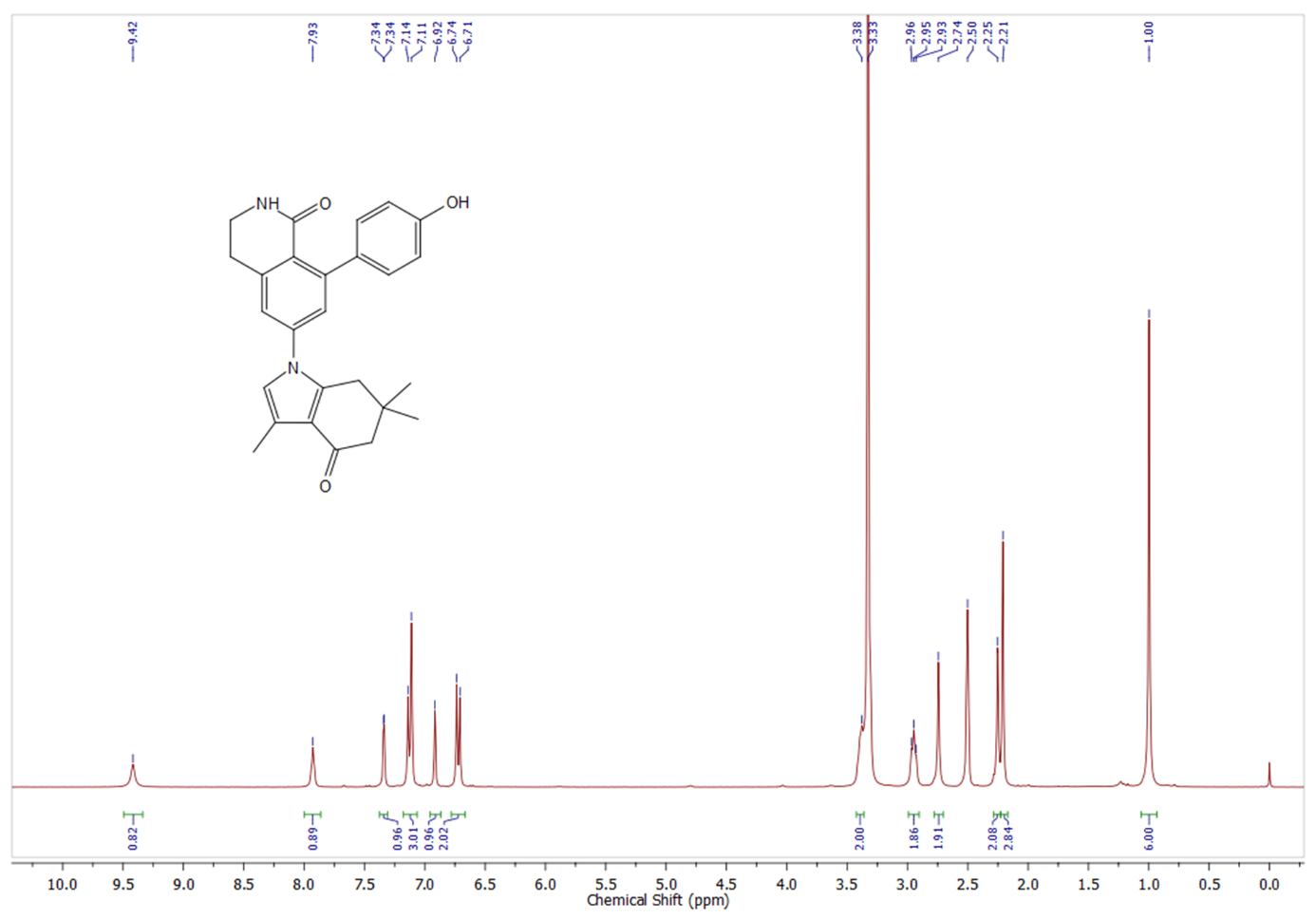
Figure S10** ^1^H spectrum of **9** (DMSO-*d*_6,_ 300 MHz).

**
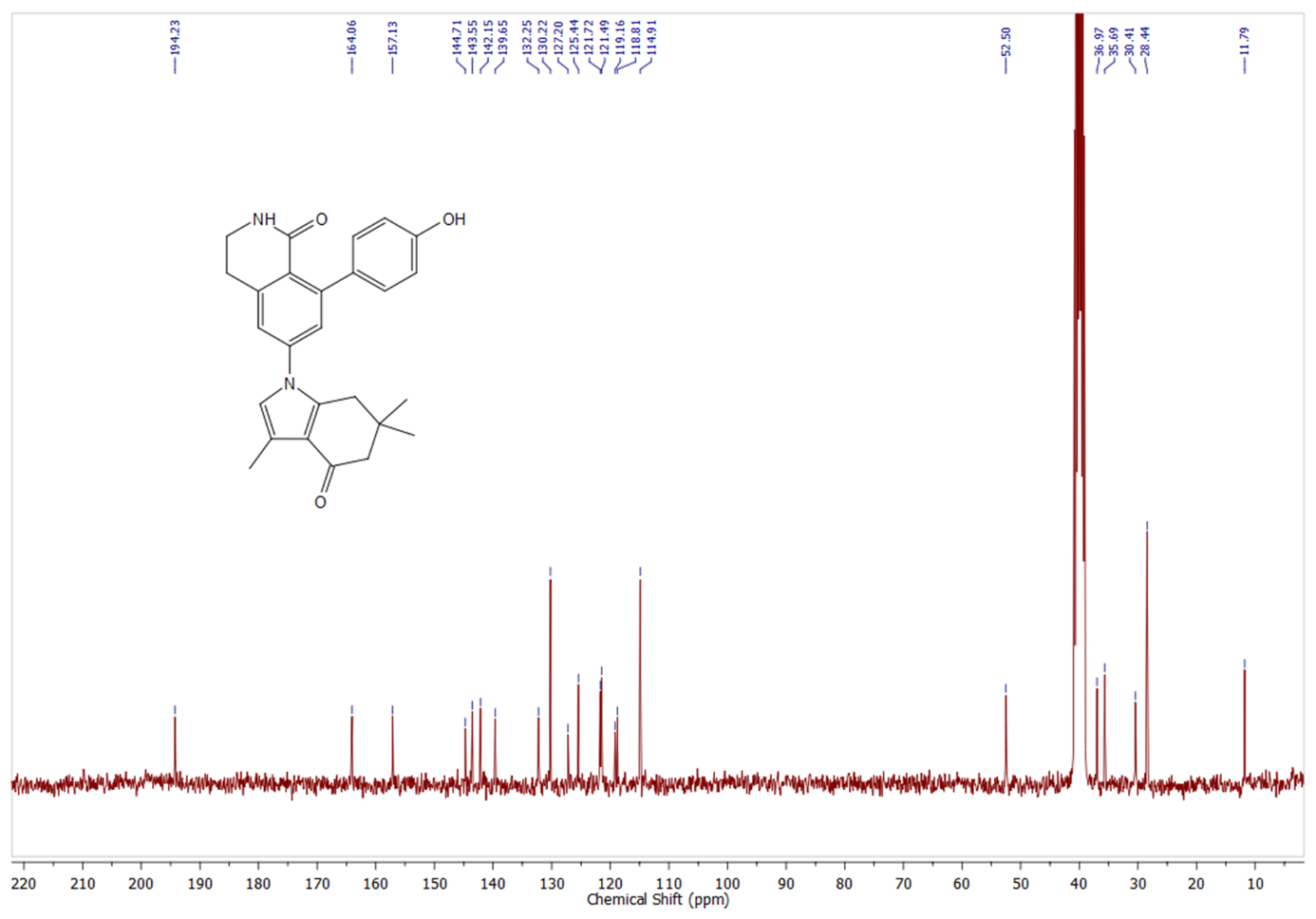
Figure S11** ^13^C spectrum of **9** (DMSO-*d*_6_, 75 MHz).

**Biological experiments**

**Table S1** HPLC gradient mixture, with A=CH_3_CN and B= NaOAc 0.05M pH 5.5, and flow rate used at given time points for the radiometabolite study of [^11^C]SNX-ab on a Chromolith RP C_18_ column.

| **Time (min)** | **A (%)** | **B (%)** | **Flow (mL/min)** |
| --- | --- | --- | --- |
| 0 | 1 | 99 | 0.5 |
| 4 | 1 | 99 | 0.5 |
| 4.1 | 1 | 99 | 1.0 |
| 9 | 90 | 10 | 1.0 |
| 12 | 90 | 10 | 1.0 |
| 12.1 | 90 | 10 | 0.5 |
| 15 | 1 | 99 | 0.5 |


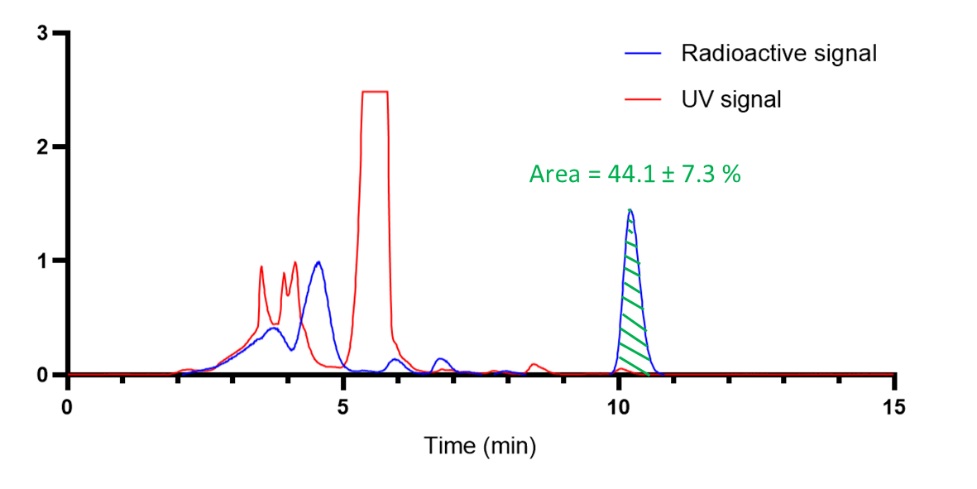


**Figure S12** Preparative HPLC chromatogram of [^11^C]SNX-ab on an XBridge RP-C_18_ column (5 µm, 4.6 mm × 150 mm). Radioactive (blue) and UV at 254 nm (red) signal are represented with indicated peak integration (green) of the carbon-11 labeled compound.

**
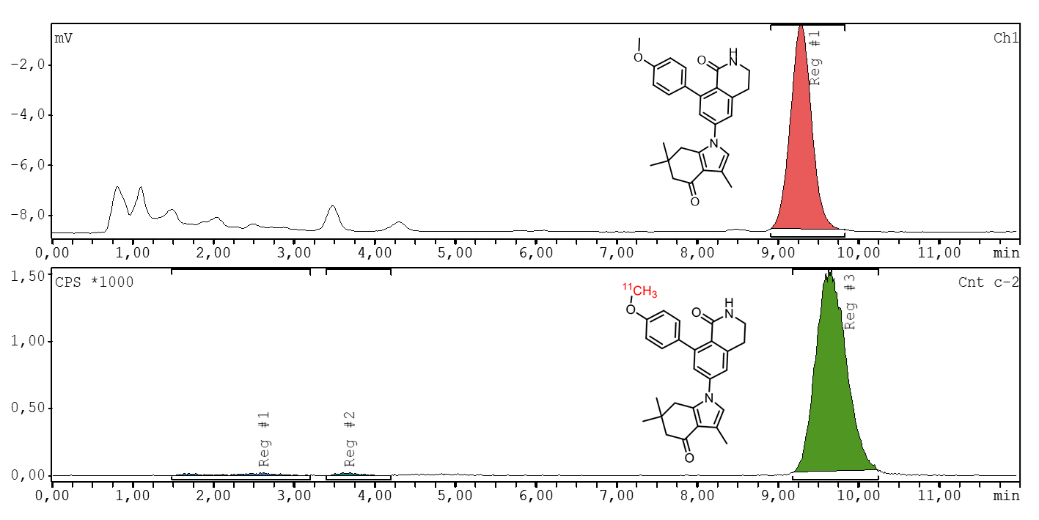
**

**Figure S13** QC HPLC chromatogram of [^11^C]SNX-ab spiked with authentic reference compound SNX-ab on an XBridge RP-C_18_ column (3.5 µm, 3.0 mm x 100 mm). The upper channel represents the UV signal at 254nm with indicated peak integration (red), corresponding to the authentic reference compound. The lower channel represents the radioactive signal with indicated peak integration (green), corresponding to the carbon-11 labeled compound.


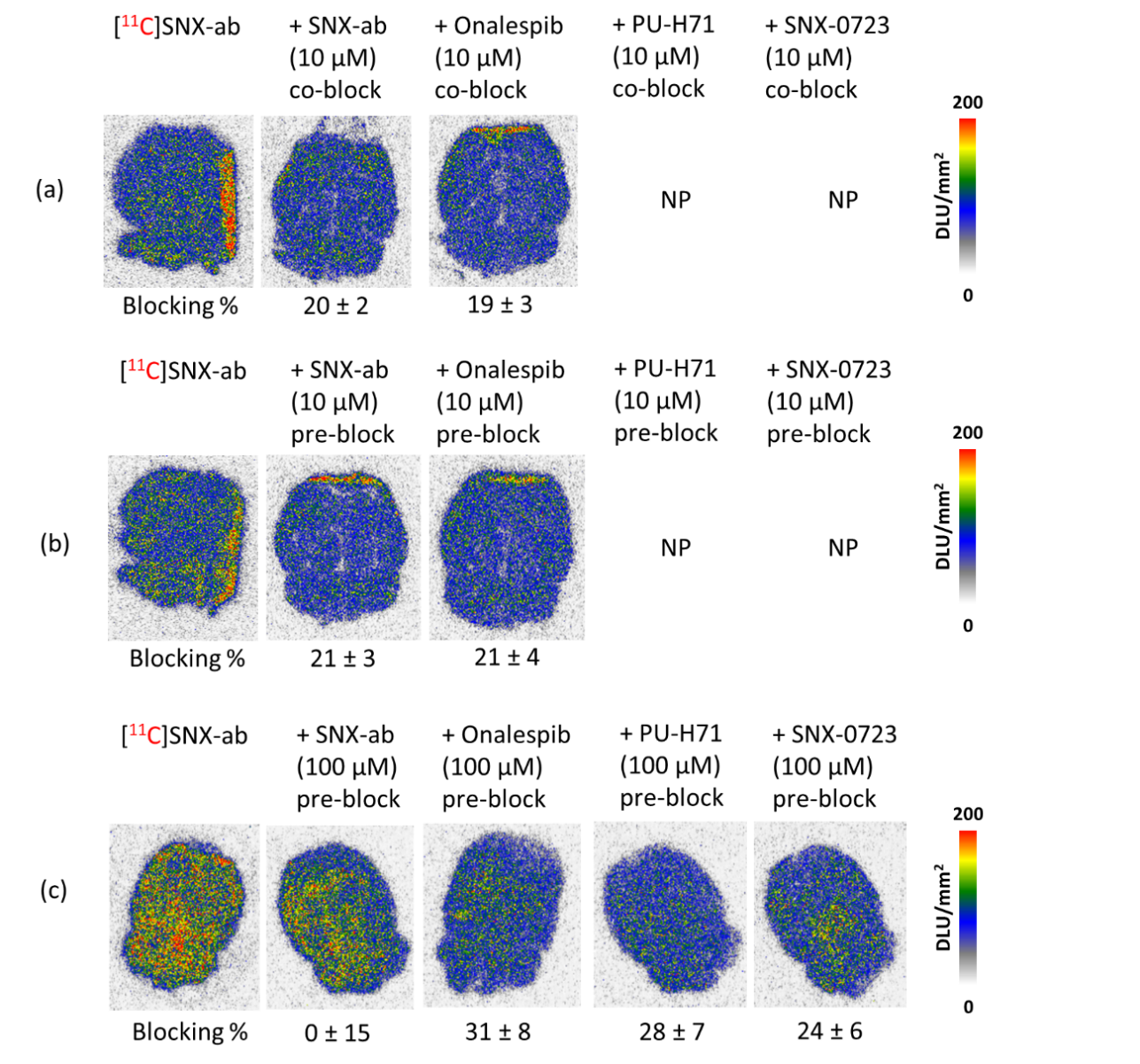


**Figure S14** In vitro autoradiography. Mouse brain slices were incubated with [^11^C]SNX-ab (74 kBq/mL). Binding specificity was assessed by (a) co-incubation with homologous (SNX-ab) and heterologous (Onalespib) inhibitors at a concentration of 10 µM (n = 4) (b) pre-incubation with homologous (SNX-ab) and heterologous (Onalespib) inhibitors at a concentration of 10 µM (n = 4) (c) pre-incubation with homologous (SNX-ab, SNX-0723) and heterologous (Onalespib, PU-H71) inhibitors at a concentration of 100 µM (n = 3). Intensity is depicted as DLU/mm^2^. Blocking% was calculated as (1-(average DLU/mm^2^ in tissue slice in the presence of blocking agent))/(average DLU/mm^2^ in tissue slice tracer only)*100%. Data are presented as mean ± SD. NP = not performed.

**Table S2** In vitro cell binding. Live U87 cells were incubated with [^11^C]SNX-ab (250 kBq/mL). Binding specificity was assessed by pre-incubation with homologous (SNX-ab, SNX-0723) and heterologous (Onalespib) inhibitors (n = 3). % of applied radioactivity was determined in the PBS-wash, Gly-wash and lysate fraction, corresponding to free radioligand, membrane-bound radioligand and intracellularly bound radioligand respectively. Data are presented as mean ± SD.

|  | **Control** | **SNX-ab** | **Onalespib** | **SNX-0723** |
| --- | --- | --- | --- | --- |
| **Intracellularly bound radioligand** | 2.0 ± 0.1 | 1.3 ± 0.1 | 1.0 ± 0.1 | 1.0 ± 0.1 |
| **Membrane-bound radioligand** | 1.6 ± 0.1 | 2.0 ± 0.1 | 1.5 ± 0.2 | 1.6 ± 0.1 |
| **Free radioligand** | 96.4 ± 0.1 | 96.6 ± 0.2 | 97.5 ± 0.3 | 97.4 ± 0.2 |


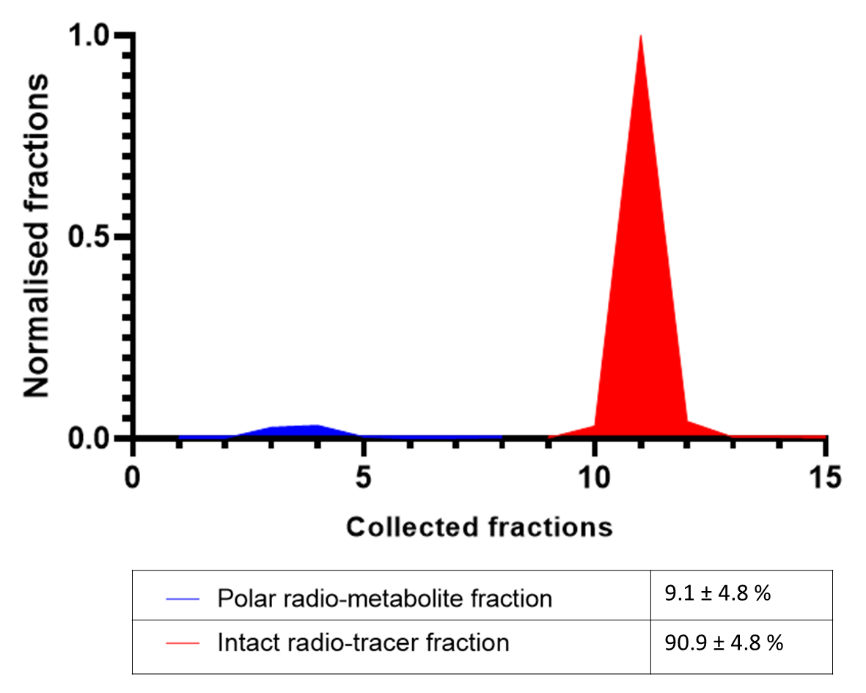


**Figure S15** Averaged radio-HPLC chromatogram of plasma radio-metabolite study on healthy C57BL/6 mice (n = 3) with indication of polar radio-metabolite and intact tracer fraction at 10 min post tracer injection. Data are presented as mean ± SD.

**Table S3** Biodistribution data of [^11^C]SNX-ab in C57BL/6 mice at 10 and 60 min post tracer injection.

|  | **%ID^a^** | | **SUV^b^** | |
| --- | --- | --- | --- | --- |
|  | **10 min** | **60 min** | **10 min** | **60 min** |
| **Urine** | 0.4 ± 0.0 | 1.0 ± 0.1 |  |  |
| **Kidneys** | 16.0 ± 1.3 | 14.6 ± 1.0 | 11.7 ± 0.6 | 12.3 ± 0.4 |
| **Liver** | 36.7 ± 2.9 | 34.7 ± 0.7 | 7.2 ± 0.1 | 7.2 ± 0.2 |
| **Intestines** | 9.0 ± 2.0 | 12.8 ± 0.8 |  |  |
| **Stomach** | 0.5 ± 0.1 | 0.5 ± 0.01 |  |  |
| **Spleen** | 0.6 ± 0.6 | 0.2 ± 0.02 | 1.0 ± 0.4 | 0.8 ± 0.1 |
| **Pancreas** | 0.7 ± 0.5 | 0.9 ± 0.2 | 1.1 ± 0.4 | 1.3 ± 0.1 |
| **Lungs** | 0.7 ± 0.2 | 0.5 ± 0.0 | 0.9 ± 0.1 | 0.9 ± 0.3 |
| **Heart** | 0.6 ± 0.1 | 0.5 ± 0.1 | 1.1 ± 0.1 | 1.1 ± 0.2 |
| **Brain** | 0.1 ± 0.0 | 0.1 ± 0.0 | 0.1 ± 0.0 | 0.1 ± 0.0 |
| **Blood** | 2.6 ± 0.3 | 2.7 ± 0.2 | 0.4 ± 0.0 | 0.4 ± 0.0 |
| **Carcass** | 32.9 ± 3.8 | 32.0 ± 1.2 |  |  |
| **Bone** | 1.6 ± 0.2 | 3.5 ± 0.4 | 0.1 ± 0.0 | 0.3 ± 0.0 |
| **Muscle** | 32.5 ± 1.9 | 19.0 ± 5.1 | 0.8 ± 0.1 | 0.5 ± 0.1 |

^a^ Percentage of injected dose dose calculated as cpm in organ/total cpm recovered) x 100. ^b^ SUV calculated as (radioactivity in cpm in organ/weight of organ in grams)/(total cpm recovered/body weight). Data expressed as mean ± SD; n = 3 per time point.

**Table S4** Percentage of radioactivity levels attributed to blood cell fraction in blood and bone marrow in bone of C57BL/6 mice at 10 and 60 min post tracer injection. Data expressed as mean ± SD; n = 3 per time point.

|  | **Blood** | **Bone** |
| --- | --- | --- |
|  | **Blood cells** | **Bone marrow** |
| **10 min** | 65.7 ± 6.9 | 87.0 ± 2.6 |
| **60 min** | ND | 72.5 ± 3.3 |
